# Encapsulation of LXR ligand by D-Nap-GFFY hydrogel enhances anti-tumorigenic actions of LXR and removes LXR-induced lipogenesis

**DOI:** 10.7150/thno.53139

**Published:** 2021-01-01

**Authors:** Ke Feng, Chuanrui Ma, Yuxin Liu, Xiaoxiao Yang, Zhimou Yang, Yaoxia Chen, Tengyan Xu, Chengbiao Yang, Shuang Zhang, Qi Li, Zhuo Wei, Dan Zhao, Peng Zeng, Jihong Han, Jie Gao, Yuanli Chen, Yajun Duan

**Affiliations:** 1 College of Life Sciences, State Key Laboratory of Medicinal Chemical Biology, Key Laboratory of Bioactive Materials of Ministry of Education, Nankai University, Tianjin, China.; 2 First Teaching Hospital of Tianjin University of Traditional Chinese Medicine, Tianjin, China.; 3 Key Laboratory of Metabolism and Regulation for Major Diseases of Anhui Higher Education Institutes, College of Food and Biological Engineering, Hefei University of Technology, Hefei, China.; 4 School of Materials Science and Engineering, Center of Functional Biomaterials, Key Laboratory of Polymeric Composite Materials and Functional Materials of Ministry of Education, GD Research Center for Functional Biomaterials Engineering and Technology, Sun Yat-sen University, Guangzhou, China.

**Keywords:** LXR, naphthylacetic acid modified D-enantiomeric-glycine-phenylalanine-phenylalanine-tyrosine (D-Nap-GFFY) hydrogel, urethane-induced pulmonary carcinomas, anti-tumor immune responses, IFNγ

## Abstract

**Background and purpose:** Activation of liver X receptor (LXR) by its ligand T0901317 (T317) enhances interferon-γ (IFNγ) production to inhibit tumor growth. However, induction of severe hypertriglyceridemia and fatty liver by T317 limits its application. The naphthylacetic acid modified D-enantiomeric-glycine-phenylalanine-phenylalanine-tyrosine (D-Nap-GFFY) can form a nanofiber hydrogel which is selectively taken up by antigen-presenting cells (APCs). In this study, we determined if D-Nap-GFFY-encapsulated T317 (D-Nap-GFFY-T317) can potently inhibit tumor growth while having no adverse lipogenic effects on the liver.

**Methods:** We prepared D-Nap-GFFY-T317 nanofiber hydrogel and subcutaneously injected it into IFNγ deficient (IFNγ^-/-^) and wild-type (WT) mice with lung carcinoma, either inoculated LLC1 cells or urethane-induced carcinoma. Mice received oral T317 administration were used for comparison. Effects of treatment on tumor growth, lipogenesis and involved mechanisms were investigated.

**Results:** Compared with T317 oral administration, injection of D-Nap-GFFY-T317 more potently inhibited LLC1 tumor growth in mice. The inhibition was dependent on LXR-activated IFNγ expression in APCs. D-Nap-GFFY-T317 increased M1 while reducing M2 type macrophages in tumors. Associated with activation of IFNγ expression, D-Nap-GFFY-T317 enhanced dendritic cell maturation and infiltration into tumors, increased CD3^+^/CD8^+^ cells in tumors, and inhibited tumor angiogenesis. Similarly, D-Nap-GFFY-T317 more potently inhibited growth of urethane-induced lung carcinomas than T317 oral administration. In these two tumor models, T317 oral administration, but not D-Nap-GFFY-T317 injection, activated hepatic lipogenesis and induced fatty liver.

**Conclusion:** Our study demonstrates that D-Nap-GFFY-T317 inhibits lung tumor growth without adverse effects on the liver, indicating the hydrogel-encapsulated LXR ligand might be a novel therapy for tumor treatment.

## Introduction

The liver X receptor (LXR) is a ligand-activated transcription factor. LXR plays an important role in cholesterol metabolism and lipogenesis. It has been identified as a potential drug target for atherosclerosis treatment 1-3. Moreover, the accumulating evidence suggests the involvement of LXR in a variety of malignancies and the potential application of LXR ligands in preclinical cancer models 4. T0901317 (T317) and GW3965 are synthetic LXR ligands. They were initially developed for atherosclerosis treatment but abandoned after preclinical studies due to that they can induce severe hypertriglyceridemia and fatty liver 5-7.

LXR includes two isoforms, LXRα and LXRβ 8. The LXR-induced fatty liver is mainly regulated by LXRα activity 5-7. However, these two isoforms exhibit a high identity in either DNA or ligand binding domain, which results in slower development of selective LXRβ modulators 9. In addition to LXRβ-specific ligands, tissue-specific ligands can also make contributions to reduction of the adverse effects in other target tissues, especially in the liver 4. For instance, GW6340 is an intestine-specific LXR ligand which can be used for treatment of small intestine cancer without effect on hepatic LXR 4, 10.

We previously reported that production of interferon gamma (IFNγ), a cytokine with a well-established role in anti-tumor immune responses 11, was significantly activated by T317 through activation of LXR. Furthermore, we determined that oral administration of T317 (mixed with food) inhibited growth of both inoculated and carcinogen-induced lung tumors in mice by activating IFNγ production 12, 13. IFNγ can be produced by multiple cells types including T cells, NK cells and antigen-presenting cells (APCs) of macrophages and dendritic cells (DCs) 14. Interestingly, IFNγ can activate macrophages to enhance macrophage functions, such as tumor cell cytotoxicity 15. IFNγ has also been demonstrated to induce the regression of tumor vasculature, which results in arrest of blood flow and subsequent collapse of tumors 16.

The naphthylacetic acid modified D-enantiomeric-glycine-phenylalanine-phenylalanine-tyrosine (D-Nap-GFFY) is a tetra-peptide, which can be synthesized by the Fmoc-solid phase synthesis method 17. It can be dissolved in PBS with a slight heating. When D-Nap-GFFY solution is cooled down to room temperature, it forms a nanofiber hydrogel which is injectable. More importantly, this hydrogel system can incorporate with either a small molecule or a large molecule of peptides/proteins, and carry the encapsulated molecules into cells/tissues. The previous studies demonstrate that D-Nap-GFFY can enhance the uptake and absorption of antigens [such as ovalbumin (OVA)] by APCs, especially by DCs, and promote DC maturation 17. In addition, it facilitates the accumulation of antigen-bearing APCs in lymph nodes, thereby promoting the transportation of antigens from the injection site to lymph nodes, providing a better opportunity to induce an effective immune response, demonstrating potent anti-tumorigenic functions of antibody 17, 18.

Based on the findings above, we hypothesized that the hydrogel-encapsulated LXR ligand may suppress lung tumors without effect on hepatic lipogenesis. It may also minimize the complications caused by activated antibody production. Therefore, in this study, we encapsulated T317 into D-Nap-GFFY to form D-Nap-GFFY-T317 nanofiber hydrogel, and determined if this system can enhance anti-tumorigenic functions of T317 while removing T317-induced adverse effects. Mechanistically, we investigated if the inhibition of tumor growth by D-Nap-GFFY-T317 is completed through activation of IFNγ production, particularly by macrophages and DCs.

## Results

### Characteristics of D-Nap-GFFY and D-Nap-GFFY-T317

**Figure 1:**
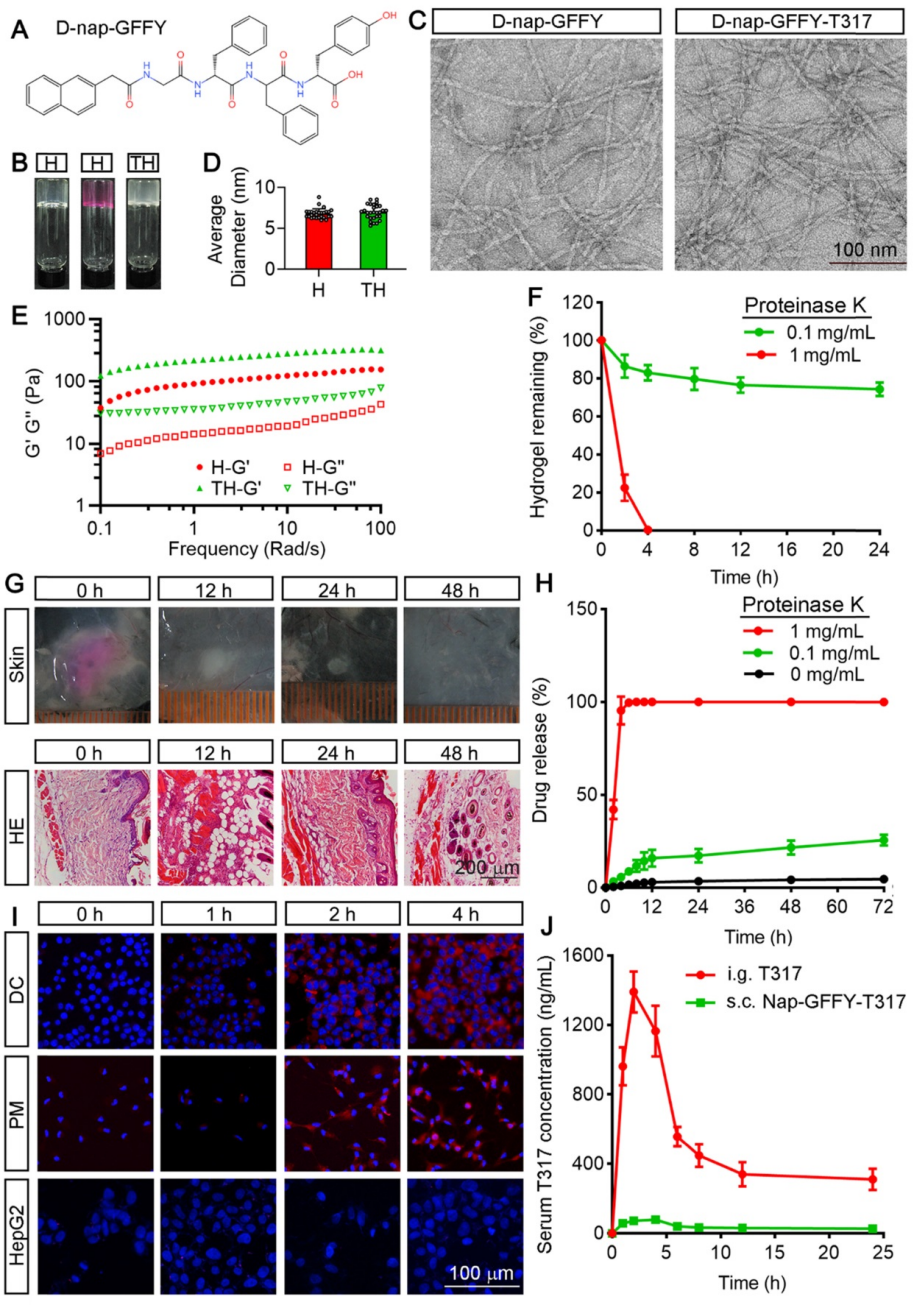
**Characterization of D-Nap-GFFY/D-Nap-GFFY-T317 and determination of T317 release from D-Nap-GFFY-T317**. (**A**) chemical structure of D-Nap-GFFY. (**B**) photographs of D-Nap-GFFY hydrogel prepared with PBS (H) and DMEM medium (H with red color), D-Nap-GFFY-T317 hydrogel prepared with PBS (TH). (**C**) the representative TEM images of D-Nap-GFFY and D-Nap-GFFY-T317. (**D**) the average diameters of D-Nap-GFFY and D-Nap-GFFY-T317 were calculated based on TEM images. (**E**) dynamic frequencey sweep of D-Nap-GFFY and D-Nap-GFFY-T317. (**F**) degradation behavior of D-Nap-GFFY under different proteinase K concentrations *in vitro*. (**G**) C57BL/6 mice were s.c. injected 100 μL D-Nap-GFFY. At the different time points after injection, mice were euthanized and the skin samples of injection site were collected, followed by photography and preparation of sections for HE staining. (**H**) the release profiles of T317 from D-Nap-GFFY-T317 in the presence of proteinase K at different concentrations *in vitro*. (**I**) DCs, peritoneal macrophages and HepG2 cells were added with D-Nap-GFFY-NR and incubated for the indicated times. After washing with PBS, accumulated D-Nap-GFFY-NR within cells were determined by a fluoresent microscopy. Scale bar: 100 μm. (**J**) C57BL/6 mice (n = 3) were i.g. administrated T317 solution at 10 mg/kg bodyweight or s.c. injected D-Nap-GFFY-T317 at the same T317 dose. After treatment, mice were sacrificed at the indicated time points and blood samples were collecetd for determination of T317 concentrations by LC-MS.

We initially synthesized D-Nap-GFFY by the standard Fmoc-solid phase peptide synthesis method. The chemical structure of D-Nap-GFFY is shown in Figure 1A. We also completed the ^1^H NMR and ^13^C NMR analysis on D-Nap-GFFY and presented the results in Figure S1A-B. We then prepared D-Nap-GFFY and D-Nap-GFFY-T317 hydrogels by the heating-cooling process. As shown in Figure 1B, D-Nap-GFFY hydrogel (named it as “H”) was transparent, while D-Nap-GFFY-T317 hydrogel (named it as “TH”) was opaque. After centrifugation, there is glue deposit at the bottom of D-Nap-GFFY-T317, which is slight whiter than D-Nap-GFFY and has a uniform texture (Figure S2A). Then we tested the concentration of T317 in the upper, middle and bottom layers of the hydrogel, and found close concentrations among them (Figure S2B). These results indicate that T317 and nanofibers are adsorbed together, and T317 is evenly distributed within the hydrogel. The mean diameter of nanofiber was determined about 6.82 nm and 6.93 nm for D-Nap-GFFY and D-Nap-GFFY-T317 (Figure 1C; S2C-D), respectively, and both of them demonstrated uniform (Figure 1D).

Next, we determined the mechanical properties of hydrogels by a rheometer. In Figure 1E, both the elastic modulus G' and the viscous modulus G" show a weak frequency dependence between 0.1-100 rad/s. The results also suggest that the encapsulation of T317 slightly improves the mechanical strength of the hydrogel, since the G' value of D-Nap-GFFY-T317 is slightly larger than that of D-Nap-GFFY.

In order to explore the biostability of D-Nap-GFFY, we incubated D-Nap-GFFY hydrogel in the presence of proteinase K at different concentrations at 37 °C. As shown in Figure 1F, we found ~80% of D-Nap-GFFY remained intact within 24 h in the presence of 0.1 mg/mL proteinase K. However, D-Nap-GFFY was completely degraded after incubation with 1 mg/mL proteinase K for 4 h. These results suggest that D-Nap-GFFY is stable in aquous phase but can be degraded within cells since the presence of proteases. Furthermore, we investigated the degration of D-Nap-GFFY *in vivo*. Because D-Nap-GFFY is pellucid and it is hard to identify its shape and size in the subcutaneous tissue, we prepared the D-Nap-GFFY hydrogel in DMEM medium instead of PBS (Figure 1B), and subcutaneous (s.c.) injecetd it to C57BL/6 mice. At 0 h, we observed the shape and size of the hydrogel in the subcutaneous tissue. At 12 h after injection, the hydrogel faded its color, became smaller and white (upper panel of Figure 1G). Meanwhile, the results of HE staining suggest that the hydrogel injection site was infiltrated by inflammatory cells (lower panel of Figure 1G). Moreover, the volume of the hydrogel was decreasing with time and disappeared at 48 h after injection. Correspondingly, the content of infiltrating cells in the hydrogel injection site was decreased significantly (Figure 1G). These results suggest that D-Nap-GFFY has a good biocompatibility and it is phagocytosed by the inflammatory cells sorrunding the subcutaneous injection sites.

We then determined the release rate of T317 from D-Nap-GFFY-T317. Interestingly, we found the release rate of T317 was only about 4% in the absence of proteinase K and about 20% in the presence of 0.1 mg/mL proteinase K after 72 h incubation. In contrast, as shown in Figure 1H, in the presence of 1 mg/mL proteinase K, the release rate of T317 was increased to 100% after 4 h incubation, suggesting that the release of T317 depends on the degradation of D-Nap-GFFY hydrogel by protease action (Figure 1H).

In order to determine if the uptake of D-Nap-GFFY hydrogel is cell type selective, particularly by APCs, we encapsulated Nile red (NR) with D-Nap-GFFY to form D-Nap-GFFY-NR. NR is a lipophilic stain that does not fluoresce in the polar liquid but fluoresces strongly in a fat-soluble environment 19. D-Nap-GFFY-NR was co-cultured with DC 2.4 cells (a murine DC line), primary peritoneal macrophages and HepG2 cells (a human hepatic cell line) for different times. As shown in Figure 1I, the fluorescence intensity of NR was increased over time and reached maximal after 4 h incubation in both DCs and macrophages (Figure 1I). Meanwhile, little NR fluorescense was found in HepG2 cells, indicating no uptake of D-Nap-GFFY-NR by the cells (Figure 1I). Furthermore, we treated mice a single dose of T317 at 10 mg/kg bodyweight either by intragastric (i.g.) administration of T317 dissolved in corn oil or by s.c. injection of D-Nap-GFFY-T317, and then determined their pharmacokinetics. We found that the peak concentration of T317 in serum was about 1400 ng/mL with oral administration at 2 h and the half-life in blood was about 6 h (Figure 1J). However, the release profile of T317 from D-Nap-GFFY-T317 injection was very low always (Figure 1J).

Taken together, the results in Figure 1 demonstrate that D-Nap-GFFY-T317 has good mechanical property, biocompatibility and stability. It can be taken up selectively by APCs, and the realease of T317 from D-Nap-GFFY-T317 is at a slow rate and in a protease-dependent manner.

### D-Nap-GFFY-T317 inhibits growth of Lewis lung carcinoma xenografts

We initially determined the effect of D-Nap-GFFY and T317 on cell viability. We found D-Nap-GFFY didn't affect the viability of peritoneal macrophages, primary hepatocytes or Lewis lung carcinoma (LLC1) cells, indicating D-Nap-GFFY is safe to cells (Figure S3A, C, E). In contrast, although T317 had no effect on the cell viability of macrophages or hepatocytes, it substantially reduced the viability of LLC1 cells in a concentration-dependent manner, which may be due to the anti-tumorigenic effect of T317 on lung cancer (Figure S3B, D, F).

**Figure 2:**
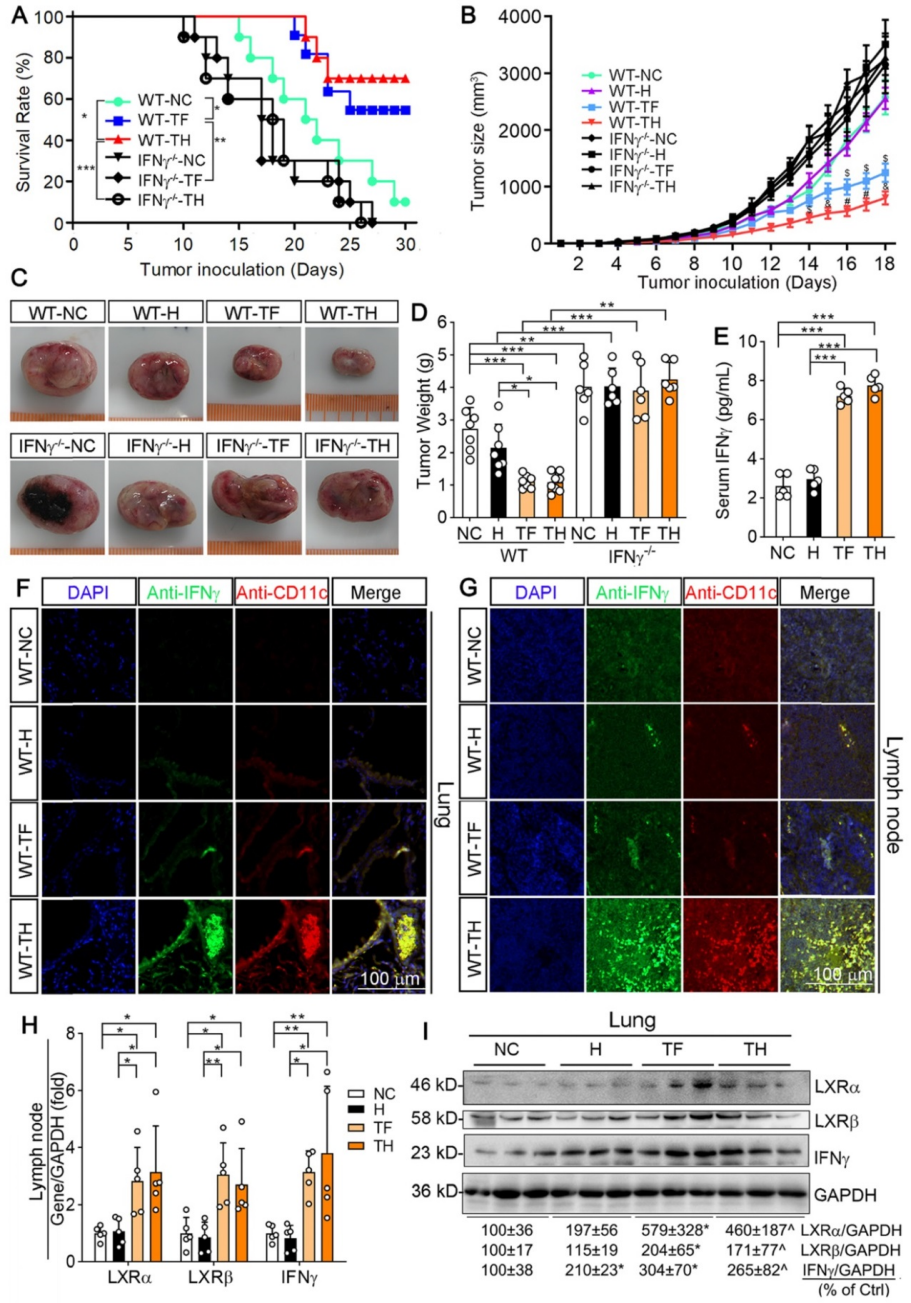
** D-Nap-GFFY-T317 inhibits growth of inoculated LLC1 tumors in an IFNγ-dependent manner.** (**A**) after s.c. injection of LLC1 cells (2 x 10^5^ cells/mouse), wild-type (WT) or IFNγ deficient (IFNγ^-/-^) were randomly divided into 3 groups (10 mice/group) and received following treatment: NC group, fed normal chow plus s.c. injection of PBS; TF group, fed normal chow containing T317 (5 mg/day/kg bodyweight); TH group, fed normal chow plus s.c. injections of D-Nap-GFFY-T317 once another day with dose of T317 at 10 mg/kg bodyweight or 5 mg/day/kg bodyweight. Mice were checked mortality daily. *P < 0.05, **P < 0.01, ***P < 0.001. (**B**) another batch of WT or IFNγ^-/-^ mice were injected LLC1 cells and then randomly divide into 4 groups. Besides the same treatment as in Figure 2**A**, one more group (H group) was included in which mice were fed normal chow and s.c. injected D-Nap-GFFY (once another day). All mice were determined tumor size daily. ^$^P < 0.05, ^&^P < 0.01, ^#^P < 0.001 *vs.* WT-NC (n ≥ 6). After 18 days of treatment, mouse blood (WT mice only), lung and tumor samples were collected individually. Tumors were photographed (**C**) and weighed (**D**), *P < 0.05, **P < 0.01, ***P < 0.001 (n ≥ 6); Serum IFNγ levels in WT mice were determined by ELISA assay (**E**), n = 5. (**F-G**) sections of lung and lymph nodes were conducted co-immunofluorescent staining with anti-IFNγ and CD11c antibodies, respectively. (**H-I**) expression of LXRα, LXRβ and IFNγ mRNA and protein were determined by qRT-PCR and Western blot, respectively. *P < 0.05; **P < 0.01; ***P < 0.001 (n = 5); *P < 0.05 *vs*. NC, ^P < 0.05 *vs*. NC and D-Nap-GFFY (n = 3).

We then determined the anti-tumorigenic properties of D-Nap-GFFY-T317 on mice inoculated with LLC1 cells. Wild-type (WT) or IFNγ deficient (IFNγ^-/-^) mice were s.c. injected LLC1 cells (2 x 10^5^ cells/mouse). Mice were then randomly divided into three groups and received following treatment: 1) NC group, mice were fed normal chow; 2) TF group, mice were fed normal chow containing T317 and the dose of T317 was estimated at ~5 mg/day/kg bodyweight; and 3) TH group, mice were fed normal chow and s.c. injected D-Nap-GFFY-T317 once another day with T317 at a dose of ~10 mg/kg bodyweight or equivalent to ~5 mg/day/kg bodyweight. Mice were checked alive or dead daily. In WT groups, one dead mouse was initially found on day 15 of treatment and 90% of mice were dead at the end of 30 days treatment in NC group. However, treatment of mice with T317 contained in food or s.c. injection of D-Nap-GFFY-T317 delayed the death of animals, and the first dead mouse was found on day 20 and 21 in TF and TH group, respectively (Figure 2A). More importantly, as shown in Figure 2A, at the end of experiment, 60% of mice were still alive by T317 treatment contained in food (TF), while 70% of mice survived by D-Nap-GFFY-T317 injection (TH). In contrast, lack of IFNγ expression resulted in earlier death in the mice. In addition, neither T317 in food nor D-Nap-GFFY-T317 had effect on IFNγ^-/-^ mouse survival rate (Figure 2A). Taken together, the results in Figure 2A indicate that encapsulation of T317 in D-Nap-GFFY can further increase the survival rate of mice inoculated LLC1 tumor cells, and the protection should be dependent on IFNγ expression.

The results above also suggest that treatment of mice with T317 in food or D-Nap-GFFY-T317 injection can inhibit tumor growth. Therefore, we used another batch of animals to determine the effect of treatment on growth of LLC1 tumors. In WT mice, compared with NC group, tumor size and weight were slightly affected by injection of D-Nap-GFFY, but they were remarkably decreased by T317 oral administration and D-Nap-GFFY-T317 injection (Figure 2B-D). Compared with WT mice, the faster tumor growth and bigger tumor size/weight were determined in control IFNγ^-/-^ mice. In addition, neither T317 in food nor D-Nap-GFFY had effect on tumor growth/weight in IFNγ^-/-^ mice (Figure 2B-D).

To link the inhibition of LLC1 tumor growth by D-Nap-GFFY-T317 to activation of IFNγ expression, we initially determined changes of serum IFNγ levels. As shown in Figure 2E, both T317 oral administration and D-Nap-GFFY-T317 injection significantly increased serum IFNγ levels. In addition, activation of IFNγ expression in lung and lymph nodes by T317 oral administration or D-Nap-GFFY-T317 injection was confirmed by immunohistochemical staining (Figure S4A-B). Meanwhile, markers of DCs and macrophages were colocalized with activated IFNγ in lung and lymph nodes (Figure 2F-G; S4C-H), respectively. To further confirm the effect of D-Nap-GFFY-T317 on DC infiltration, the adjacent tissue slices of the same tissues (lung and lymph nodes) or tumors were used to determine expression of two related markers of DCs, LY75 and CD11c, by co-immunofluorescent staining with anti-LY75 plus anti-IFNγ, or anti-CD11c plus anti-IFNγ antibodies. And the results in Figure S5 suggest that D-Nap-GFFY-T317 significantly promotes APCs infiltration as well as IFNγ expression in tumor, lung and lymph nodes.

Associated with activation of IFNγ protein and mRNA expression, D-Nap-GFFY-T317 injection induced expression of LXRα and LXRβ mRNA and protein at a comparable level as T317 oral administration in mouse tissues (Figure 2H-I), which might be due to the presence of infiltrating APCs, particularly monocytes, after uptake of D-Nap-GFFY-T317. It also demonstrates that D-Nap-GFFY-T317 can function similarly to free T317 to activate LXR and subsequent IFNγ expression, thereby inhibiting tumor growth in the IFNγ-dependent manner.

### D-Nap-GFFY-T317 activates IFNγ expression in APCs and DC maturation, but suppresses angiogenesis

**Figure 3:**
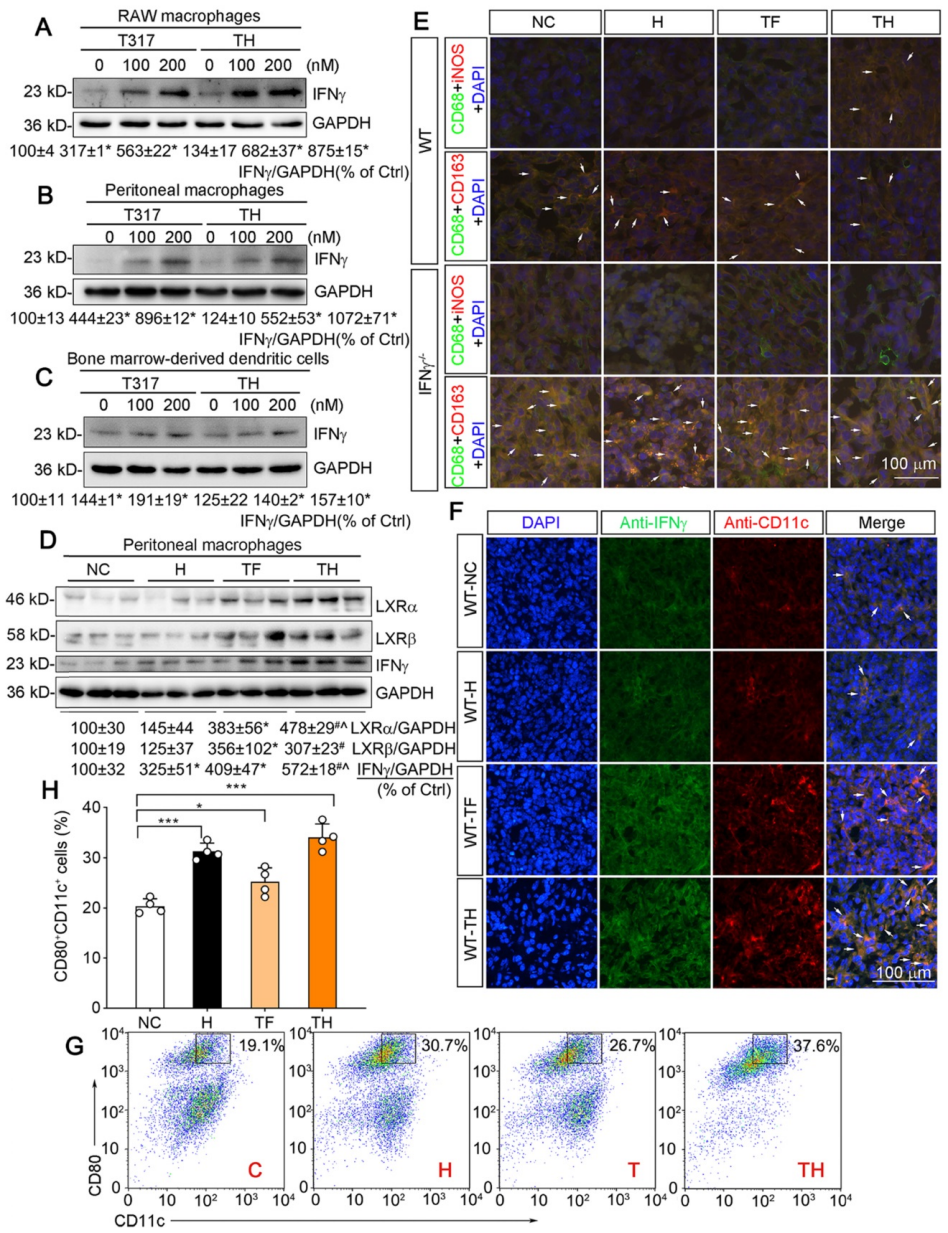
** D-Nap-GFFY-T317 induces APC IFNγ expression, macrophage type I polarization and DC infiltration/maturation in tumors.** (**A-C**) RAW264.7 macrophages, mouse peritoneal macrophages and mouse bone marrow-derived DCs were treated with T317 or D-Nap-GFFY-T317 at the indicated concentrations for 16 h. (**D**) peritoneal macrophages were isolated from WT mice used in Figure 2**B-D**. Expression of IFNγ, LXRα and LXRβ were determined by Western blot. (**E**) tumor sections prepared from WT and IFNγ^-/-^ mice used in Figure 2**B-D** were conducted co-immunofluorescent staining with anti-iNOS or CD163 antibody and anti-CD68 antibodies. The arrows indicate M1 (CD68^+^iNOS^+^) or M2 (CD68^+^CD163^+^) type macrophages in tumor sections. (**F**) tumor sections from WT mice used in Figure 2**B-D** were conducted co-immunofluorescent staining with anti-IFNγ and CD11c antibodies. The arrows indicate IFNγ expressing DCs. (**G-H**) bone marrow-derived DCs were incubated with medium (C, Control), D-Nap-GFFY hydrogel (H, 50 μg/mL), T317 (T, 200 nM) or D-Nap-GFFY-T317 (TH) for 16 h at 37 °C. CD11c^+^CD80^+^ cells were determined by FACS assay. *P < 0.05; **P < 0.01; ***P < 0.001 (n = 4).

APCs including macrophages and DCs are the major IFNγ-producing cell types. We previously reported that LXR activation can induce macrophage IFNγ expression 12. To determine the effect of D-Nap-GFFY-T317 on IFNγ expression in APCs, we initially treated RAW264.7 macrophages (a murine macrophage cell line) with T317 solution or D-Nap-GFFY-T317, and observed that IFNγ expression was increased by both in a concentration-dependent manner (Figure 3A). Similarly, D-Nap-GFFY-T317 induced IFNγ expression in mouse peritoneal macrophages at the comparable level as T317 (Figure 3B). Next, we treated matured DCs with T317 and D-Nap-GFFY-T317. Similar to macrophages, IFNγ expression in DCs was also increased by T317 and D-Nap-GFFY-T317 in a concentration-dependent manner (Figure 3C).

Similar to the results in *in vitro* studies (Figure 3A-C), we collected peritoneal macrophages from mice at the end of experiment, and determined that T317 and D-Nap-GFFY-T317 induced IFNγ expression with a greater effect by D-Nap-GFFY-T317 in the cells. Associated with induction of IFNγ expression, expression of LXRα and LXRβ in mouse peritoneal macrophages was also activated by T317 or D-Nap-GFFY-T317 (Figure 3D).

Macrophage polarization can affect tumor development. As shown in Figure 3E, D-Nap-GFFY-T317 treatment resulted in more M1 type but fewer M2 type macrophages in tumors of WT mice, while IFNγ deficiency blocked the switch of macrophage cell types by D-Nap-GFFY-T317 (Figure 3E; S4I-J), suggesting the function of D-Nap-GFFY-T317 on macrophage polarization in tumors is also IFNγ-dependent.

It has been reported that D-peptide hydrogel can promote DC infiltration/maturation 17. In this study, we also assumed that D-Nap-GFFY-T317 can have a positive impact on maturation of mouse DCs. We initially determined the effect of treatment on CD11c expression in tumor sections by immunofluorescent staining. As shown in Figure 3F and S4K, associated with activated IFNγ expression, CD11c expression in tumors was also significantly increased by T317 oral administration and D-Nap-GFFY-T317 injection with a greater effect by D-Nap-GFFY-T317, indicating D-Nap-GFFY-T317 enhances the infiltration of DCs into tumors. The effect of D-Nap-GFFY-T317 on DC infiltration was further confirmed by co-immunofluorescent staining of adjacent tissue slices of tumors with anti-LY75 (another DC marker) and IFNγ antibodies, or anti-CD11c and IFNγ antibodies (Figure S5C).

*In vitro*, treatment of bone marrow (BM)-derived DCs with D-Nap-GFFY, T317 or D-Nap-GFFY-T317 enhanced DC maturation with the greatest effect by D-Nap-GFFY-T317 (Figure 3G-H).

**Figure 4:**
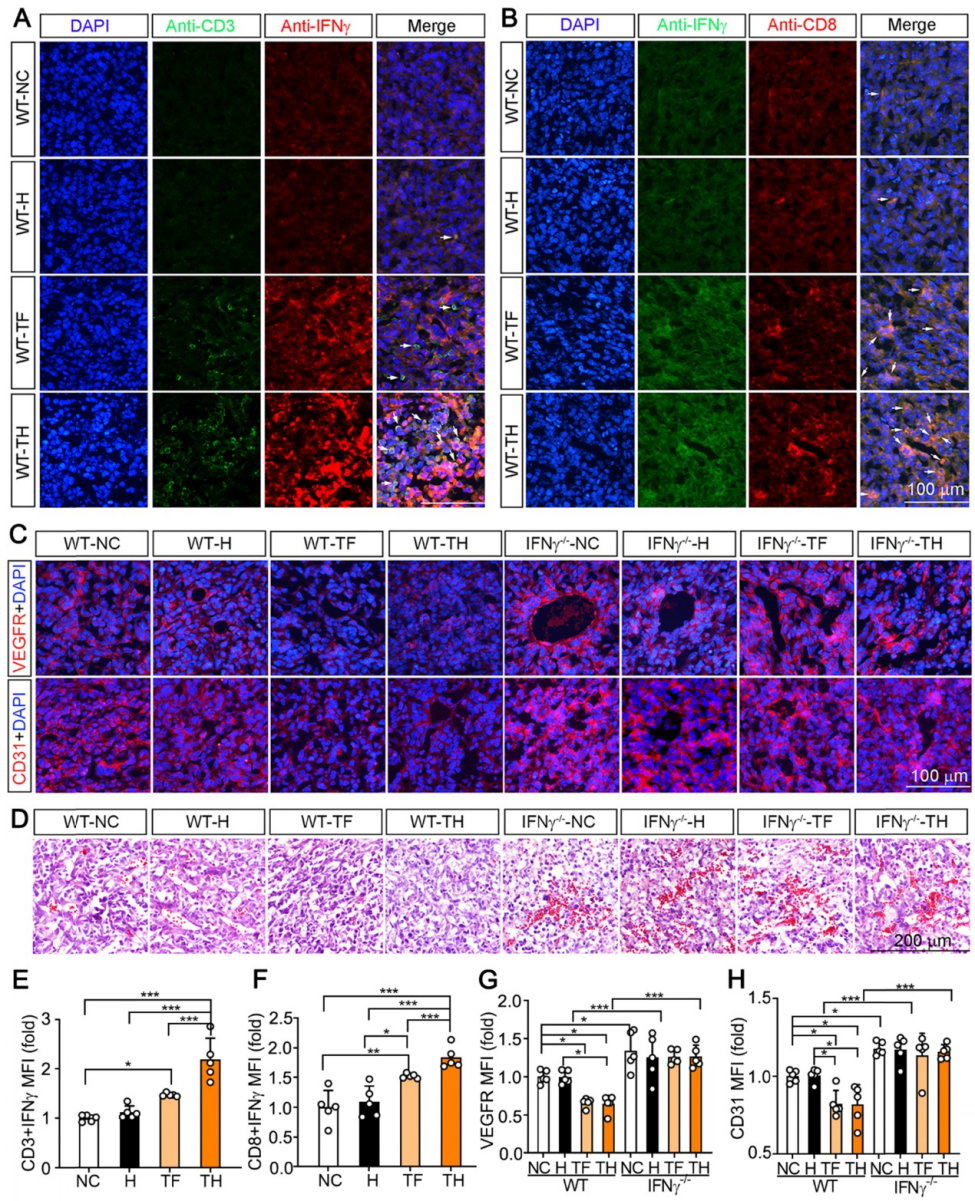
** D-Nap-GFFY-T317 induces T lymphocyte IFNγ expression and suppresses angiogenesis.** The tumor sections from WT mice used in Figure 2**B-D** were conducted co-immunofluorescent staining with anti-IFNγ and CD3 (for T lymphocytes, **A**) or CD8 (for CTLs, **B**) antibodies. The white arrows indicate IFNγ expressing T lymphocytes or CTLs. (**C**) VEGFR or CD31 expression in tumor sections were determined by immunofluorescent staining with anti-VEGFR or CD31 antibody. (**D**) HE staining of tumor sections. (**E-H**) the statistical results of mean fluorescent intensity (MFI) in images of (**A-C**) were quantified. *P < 0.05; **P < 0.01; ***P < 0.001 as indicated (n = 5).

Infiltrating and activated DCs can express and secrete IFNγ, which further promotes activation of cytotoxic T lymphocytes (CTLs) and infiltration of the circulating CTLs into tumors to destroy malignant cells 20. In this study, compared with other groups, more CD3^+^ and CD8^+^ T cells expressing IFNγ were determined in tumors of D-Nap-GFFY-T317 (TH)-treated group (Figure 4A-B, E-F), indicating more T lymphocytes and CTLs infiltrating into tumor microenvironment with activated IFNγ expression.

Previous studies demonstrated IFNγ can induce the degeneration of tumor vasculature 16. To determine if D-Nap-GFFY-T317 can inhibit angiogenesis in tumor microenvironment, we detected expression of VEGFR and CD31 in tumors. In WT mice, expression of both VEGFR and CD31 was significantly suppressed in tumors by T317 oral administration or D-Nap-GFFY-T317 injection (left half of Figure 4C, G or H). In contrast, lack of IFNγ expression enhanced expression of VEGFR and CD31 in tumors, indicating the importance of IFNγ expression in suppressing tumor angiogenesis. In addition, the inhibitory effect of T317 oral administration or D-Nap-GFFY-T317 injection on expression of VEGFR and CD31 in WT mice was attenuated in the absence of IFNγ expression (right half of Figure 4C, G or H). Consistently, the results of HE staining of tumor sections confirm the anti-angiogenesis function of T317 or D-Nap-GFFY-T317 in tumor microenvironment, which is also in an IFNγ-dependent manner (Figure 4D).

Taken together, the results above suggest that T317 oral administration or D-Nap-GFFY-T317 injection can promote IFNγ expression in macrophages, DCs and CTLs, and D-Nap-GFFY-T317 injection can increase infiltration of DCs and CTLs in tumors, the important mechanisms by which D-Nap-GFFY-T317 inhibits tumor growth.

### D-Nap-GFFY-T317 does not induce hepatic lipid accumulation in LLC1-inoculated mice

**Figure 5:**
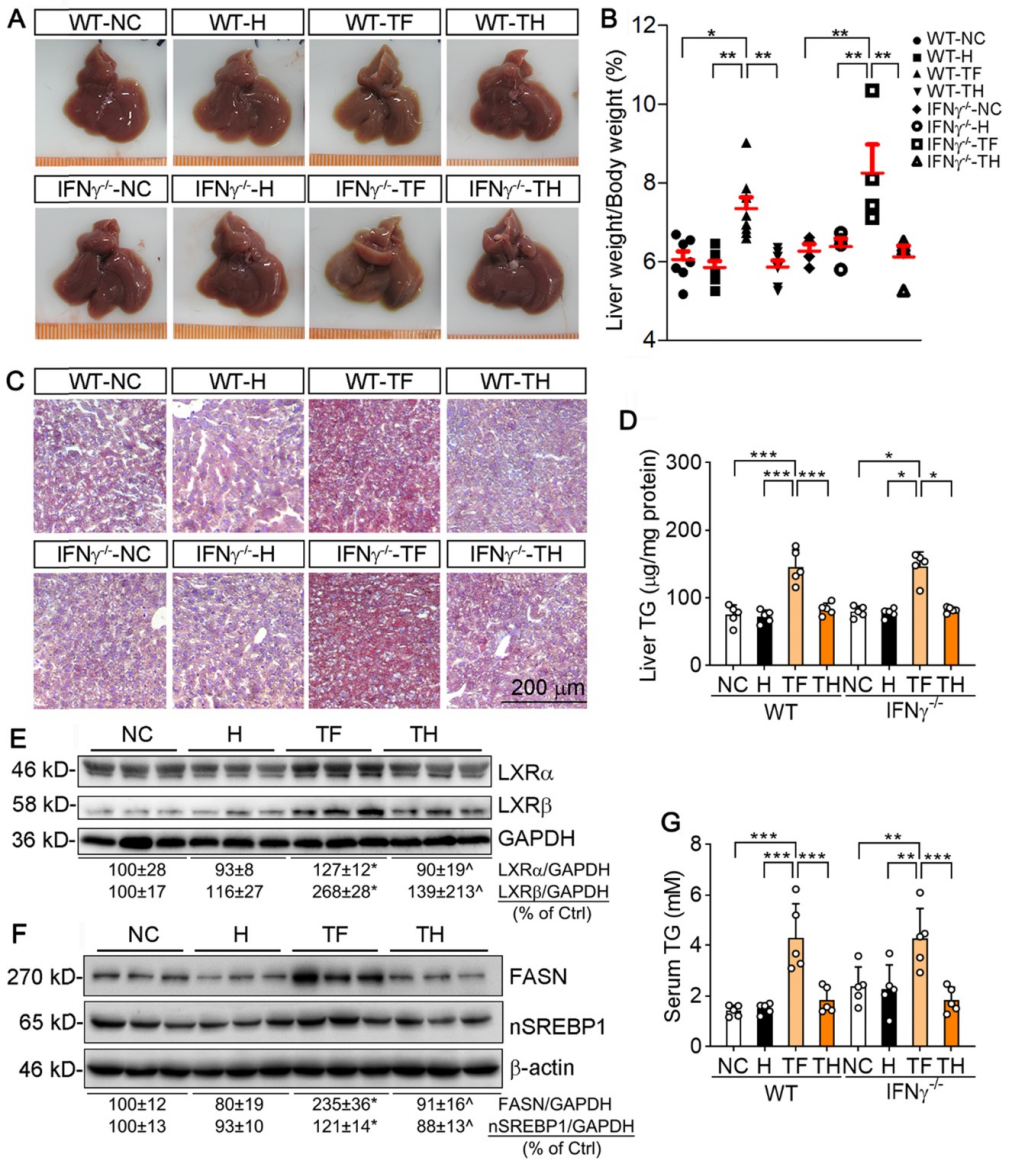
** D-Nap-GFFY-T317 protects both WT and IFNγ^-/-^ mice against T317-induced hepatic lipid accumulation.** At the end of treatment, serum and liver samples collected from mice used in Figure 2**B-D** were used to conduct the following assays. (**A**) liver photos. (**B**) ratio of liver weight to body weight. (**C**) Oil Red O staining of liver frozen sections. (**D**) TG quantitative analysis with total liver lipid extract (n = 5). Expression of LXRα and LXRβ (**E**), FASN and nSREBP1 (**F**) protein in the liver was determined by Western blot. (**G**) serum TG levels were determined using an assay kit (n = 5). *P < 0.05, **P < 0.01, ***P < 0.001 *vs*. control or as indicated, ^P < 0.05 *vs*. TF.

Activation of LXR by T317 induces severe hepatic lipogenesis and fatty liver 13. Our results showed that encapsulated NR by D-Nap-GFFY was not taken up by HepG2 cells (Figure 1I). Therefore, we speculated that D-Nap-GFFY-T317 injection can remove the adverse lipogenic effects of T317. We firstly checked mouse liver color and found that T317 oral administration reduced liver color while increasing the ratio of liver weight to body weight. In contrast, injection of D-Nap-GFFY-T317 or D-Nap-GFFY hydrogel had no effect on either liver color or size (top panel of Figure 5A, left half of B). Interestingly, the similar results were obtained with IFNγ^-/-^ mice (bottom panel of Figure 5A, right half of B), indicating the anti-lipogenic effects of D-Nap-GFFY-T317 is independent of IFNγ expression.

We then assessed the hepatic lipid content. The results of Oil Red O staining of liver sections indicate that T317 oral administration, but not D-Nap-GFFY-T317 injection, induced hepatic lipid accumulation in both WT and IFNγ^-/-^ mice (Figure 5C). The quantitative analysis of triglyceride (TG) content in total lipid extract of liver samples demonstrates that T317 oral administration increased liver TG levels while D-Nap-GFFY-T317 injection had no effect on it (Figure 5D).

To unveil the mechanisms by which T317 in food and D-Nap-GFFY-T317 influence hepatic lipid levels differently, we determined expression of LXRα and LXRβ in the liver. As shown in Figure 5E, T317 oral administration potently increased expression of LXRα and LXRβ in the tissue, while D-Nap-GFFY-T317 injection had little effect on either of them. Fatty acid synthase (FASN) and sterol-responsive element binding protein 1 (SREBP1), two important molecules responsible for fatty acid synthesis, can be activated by LXR. Correspondingly, expression of FASN and nSREBP1 (activated form of SREBP1) in the liver was substantially increased by T317 oral administration. In contrast, D-Nap-GFFY-T317 injection had little effect on both FASN and SREBP1 (Figure 5F).

Serum TG levels were also increased by T317 oral administration, not by D-Nap-GFFY-T317 injection, in both WT and IFNγ^-/-^ mice (Figure 5G). Moreover, T317 oral administration increased levels of total-cholesterol, LDL-cholesterol, alanine aminotransferase (ALT) and alkaline phosphatase (ALP) in serum of both WT and IFNγ^-/-^ mice (Table 1). In contrast, D-Nap-GFFY-T317 injection did not change these parameters either (Table 1), indicating it blocks T317-induced lipogenesis and other liver injuries.

### D-Nap-GFFY-T317 inhibits urethane-induced pulmonary carcinomas

**Figure 6:**
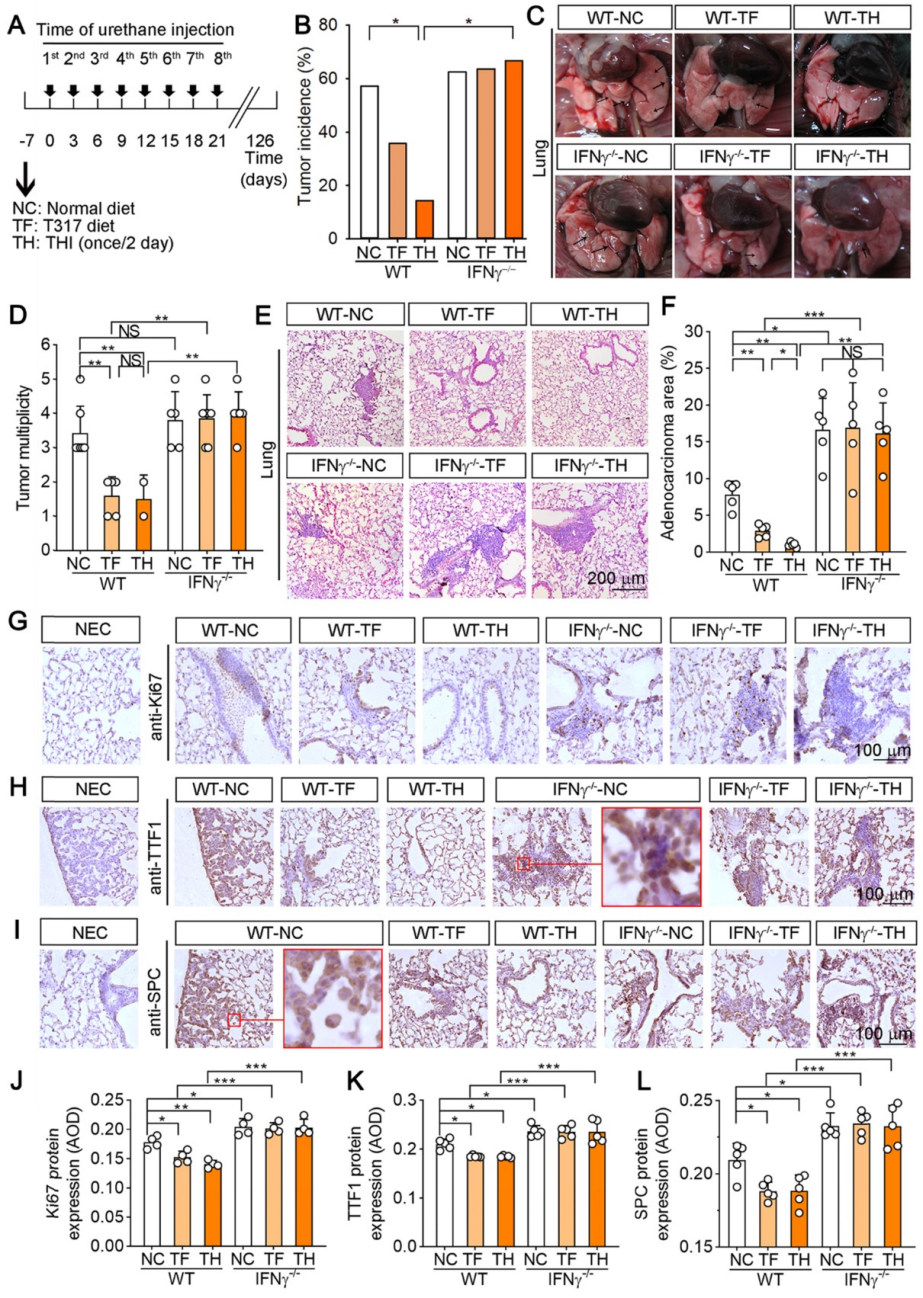
** D-Nap-GFFY-T317 inhibits formation of urethane-induced lung tumors and atypical hyperplasia in WT but not IFNγ^-/-^ mice.** (**A**) WT or IFNγ^-/-^ mice were randomly divided into 3 groups, and received following treatment: NC group, fed normal chow; TF group, fed normal chow containing T317 (5 mg/day/kg bodyweight); TH group, fed normal chow and s.c. injected D-Nap-GFFY once another day with dose of T317 at 10 mg/kg bodyweight or 5 mg/day/kg bodyweight. After one week of treatment, all the mice were i.p. injected urethane (1 g/kg bodyweight) once every 3 days for 8 times. After 126 days of the 1^st^ time urethane injection, all mice were sacrificed followed by collection of lung samples. (**B**) all lung samples were checked the tumor incidence on lung surface. (**C-D**) lung was photographed, the number of macroscopic external pulmonary nodules was counted. Arrows indicate the representative tumors. (**E-F**) after preparation, lung sections were conducted HE staining to determine tumor area with quantitative assay as % of whole section. (**G-L**) lung sections were conducted IHC staining to determine expression of Ki-67, TTF-1 and SPC with quantitative analysis of the average optical density (AOD) value (n ≥ 4). NEC: negative control, normal IgG was used to replace primary antibody. *P < 0.05, **P < 0.01, ***P < 0.001; NS: not significantly different. n = 14 for WT mice, n ≥ 8 for IFNγ^-/-^ mice as indicated in Table 2.

We also assessed the effect of D-Nap-GFFY-T317 on urethane-induced pulmonary carcinomas, the model faithfully recapitulates human lung adenocarcinoma associated with tobacco smoking 21. WT and IFNγ^-/-^ mice were randomly divided into 3 groups (10~15 mice/group) and received treatment as scheduled in Figure 6A. At the end of experiment, we found one mouse died in WT mice while 3 mice were dead in IFNγ^-/-^ mice (Table 2), which indicates the protection of IFNγ expression against pulmonary carcinomas-induced death. Although T317 oral administration and D-Nap-GFFY-T317 injection did not affect the death rate, they reduced tumor incidence significantly (Figure 6B). Furthermore, WT mice treated with T317 or D-Nap-GFFY-T317 had reduced tumors in the lung (Figure 6C-D). Compared with WT mice, IFNγ^-/-^ mice had higher tumor incidence (Figure 6B). In addition, no difference of tumor incidence or multiplicity was determined among the three groups of IFNγ^-/-^ mice (Figure 6B-D). Thus, inhibition of urethane-induced pulmonary carcinomas by T317 oral administration or D-Nap-GFFY-T317 injection also depends on activation of IFNγ expression.

The results of HE staining on lung sections show that urethane treatment induced adenomas (AD) formation in both WT and IFNγ^-/-^ mice (Figure 6E). The crowded alveolar epithelial cells and alveolar septa surrounded by anisokaryosis and karyomegaly cells were determined, indicating formation of atypical adenomatous hyperplasia (AAH). Compared with NC group, there were no AD and reduced AAH areas in WT mice after T317 oral administration or D-Nap-GFFY-T317 injection with the greater effect by D-Nap-GFFY-T317 injection (Figure 6E). Correspondingly, the area occupied by dense cellular masses and cellular atypia was decreased more by D-Nap-GFFY-T317 injection than T317 oral administration in WT mice (Figure 6F). However, neither of T317 oral administration or D-Nap-GFFY-T317 had effect on the development of AD or AAH in IFNγ^-/-^ mice (Figure 6E-F).

Next, we determined Ki-67 expression by IHC staining of lung sections, and found that there were many proliferating cells in AD areas in NC group of WT mice, and the number of proliferating cells was visually decreased by T317 oral administration in WT mice. Notably, the proliferating cells were rarely found in WT mice by D-Nap-GFFY-T317 injection (Figure 6G, J), indicating it has more potent anti-proliferative actions than T317 oral administration. Moreover, we confirmed it by detecting expression of thyroid transcription factor 1 (TTF-1) and surfactant protein C (SPC), the markers of lung tumors, in AD or AAH (Figure 6H-I, K-L). The enlarged images showed that TTF-1 is expressed in the nucleus of type II alveolar epithelial cells while SPC is expressed in the cytoplasm. Expression of TTF-1 and SPC was reduced in WT mice by T317 oral administration, and the reduction was further enhanced by D-Nap-GFFY-T317 injection. However, neither T317 oral administration nor D-Nap-GFFY-T317 injection had effect on high expression of SPC or TTF-1 in lung sections of IFNγ^-/-^ mice (Figure 6H-I, K-L).

### Inhibition of urethane-induced pulmonary carcinomas by D-Nap-GFFY-T317 depends on activation of IFNγ production

**Figure 7:**
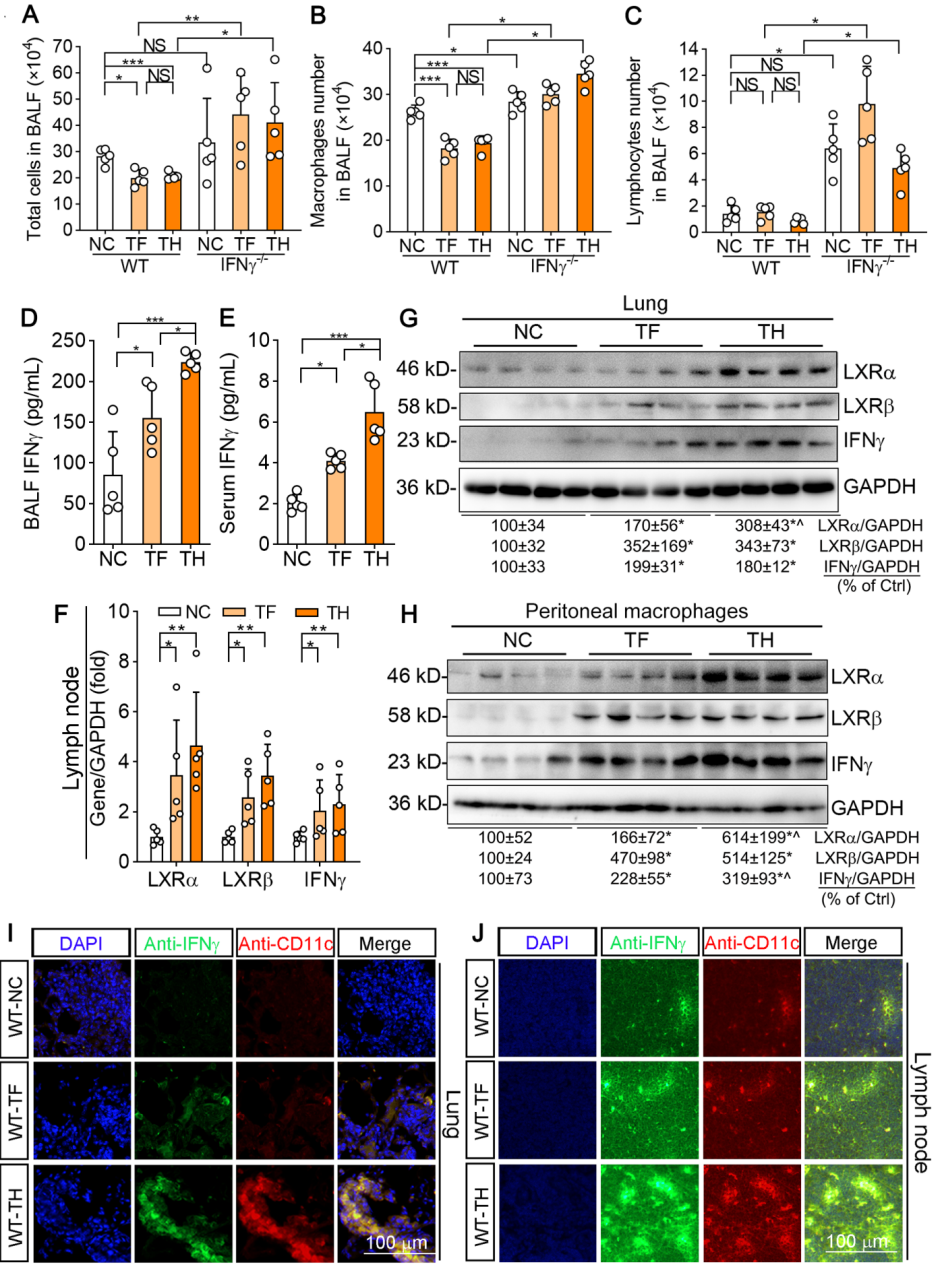
** D-Nap-GFFY-T317 increases IFNγ production and inhibits inflammation in urethane-treated WT mice but not IFNγ^-/-^ mice.** At the end of experiment, bronchoalveolar lavage fluid (BALF), blood, peritoneal macrophages, lymph nodes and lung samples were collected from WT and IFNγ^-/-^ mice used in Figure 6. (**A-C**) the number of total immune cells, macrophages and lymphocytes in BALF were counted. (**D-E**) IFNγ concentrations in BALF and serum were determined using an ELISA assay kit. (**F**) total RNA was extracted from lymph nodes followed by determination of LXRα, LXRβ and IFNγ mRNA expression by qRT-PCR. (**G-H**) expression of LXRα, LXRβ and IFNγ protein in mouse peritoneal macrophages and lung was determined by Western blot. (**I-J**) lung and lymph node sections were conducted co-immunofluorescent staining with anti-IFNγ and CD11c antibodies with the quantitative analysis of MFI presented in Figure S6**C-D**. (**A-F**): *P < 0.05, **P < 0.01, ***P < 0.001, NS: not significantly different; (**G-H**): *P < 0.05 vs. Control; ^P < 0.05 *vs*. TF, n = 4.

Several recent studies indicate that urethane-induced pulmonary inflammation can trigger the initiation of lung cancer and enhance its development 22, 23. The previous study also demonstrates that lymphocytes and macrophages are increased in the bronchoalveolar lavage fluid (BALF) of NF-κB1 KO mice, suggesting NF-κB1 restricts urethane-induced pro-tumorigenic inflammation 23. The cell number in BALF can reflect the state of pro-tumorigenic inflammation. To investigate whether the tumor-suppression by T317 or D-Nap-GFFY-T317 was related to reduce inflammation, we examined the numbers and kinds of cell types in BALF. In fact, total cells presented in BALF were reduced by T317 oral administration or D-Nap-GFFY-T317 injection in WT mice (Figure 7A). Further analysis shows reduced cells in BALF by T317 oral administration or D-Nap-GFFY-T317 injection were macrophages, not lymphocytes (Figure 7B-C). In contrast, more total cells, macrophages and lymphocytes in BALF were determined in control IFNγ^-/-^ mice, and neither T317 oral administration nor D-Nap-GFFY-T317 injection was able to reduce them (Figure 7A-C). These results suggest that T317 or D-Nap-GFFY-T317 reduces pro-tumorigenic inflammation in WT mice.

Although the cell number of macrophages was decreased by T317 or D-Nap-GFFY-T317, surprisingly, IFNγ levels in BALF were increased significantly (Figure 7D). Meanwhile, serum IFNγ level in T317- or D-Nap-GFFY-T317-treated group was substantially increased compared with NC group. In addition, higher serum IFNγ levels were determined in D-Nap-GFFY-T317-treated group than T317-treated group, indicating D-Nap-GFFY-T317 further enhanced circulating IFNγ (Figure 7E). Associated with activation of LXRα and LXRβ, both T317 oral administration and D-Nap-GFFY-T317 injection increased IFNγ expression at mRNA and protein levels in lymph nodes of urethane-injected WT mice (Figure 7F; S6A). Similarly, expression of LXRα, LXRβ and IFNγ in the lung was also activated by T317 and D-Nap-GFFY-T317 (Figure 7G; S6B). In addition, both T317 oral administration and D-Nap-GFFY-T317 injection induced expression of LXRα, LXRβ and IFNγ in peritoneal macrophages isolated from urethane-injected WT mice with the greater effect by D-Nap-GFFY-T317 (Figure 7H). Consistent with the results in LLC1 xenografts, markers of DCs and macrophages colocalized with activated IFNγ in lung and lymph nodes of mice received urethane and D-Nap-GFFY-T317 injection (Figure 7I-J; S6C-D, S6E-H), suggesting that D-Nap-GFFY-T317 significantly promotes DCs and macrophages infiltration as well as IFNγ expression in lung and lymph nodes. To further confirm the effect of D-Nap-GFFY-T317 on DC infiltration, we conducted co-immunofluorescent staining with anti-LY75 plus anti-IFNγ, or anti-CD11c plus anti-IFNγ antibodies on adjacent tissue slices of the same tissue and found the co-localization of these three molecules (Figure S7). These results show that the inhibitory effect of D-NAP-GFFY-T317 on carcinogen-induced pulmonary carcinomas is related to its potent activation of IFNγ production and functions.

### Long-term D-Nap-GFFY-T317 treatment has no effect on hepatic lipogenesis and plasma lipid profiles in urethane-injected mice

As shown in Figure S8, the long-term treatment of T317 in food or injection of D-Nap-GFFY-T317 had little effect on animal body weight or the ratio of spleen weight to body weight in both urethane-injected WT and IFNγ^-/-^ mice. However, as shown in Table 3, after ~4 months treatment, T317 in food increased serum total cholesterol and LDL-cholesterol levels in both WT and IFNγ^-/-^ mice while aspartate aminotransferase (AST) activity was also increased. In contrast, the long-term D-Nap-GFFY-T317 injection had no effect on either lipid profiles or activity of aminotransferase (Table 3), suggesting the long-term D-Nap-GFFY-T317 treatment can block T317-induced hypertriglyceridemia and fatty liver.

**Figure 8:**
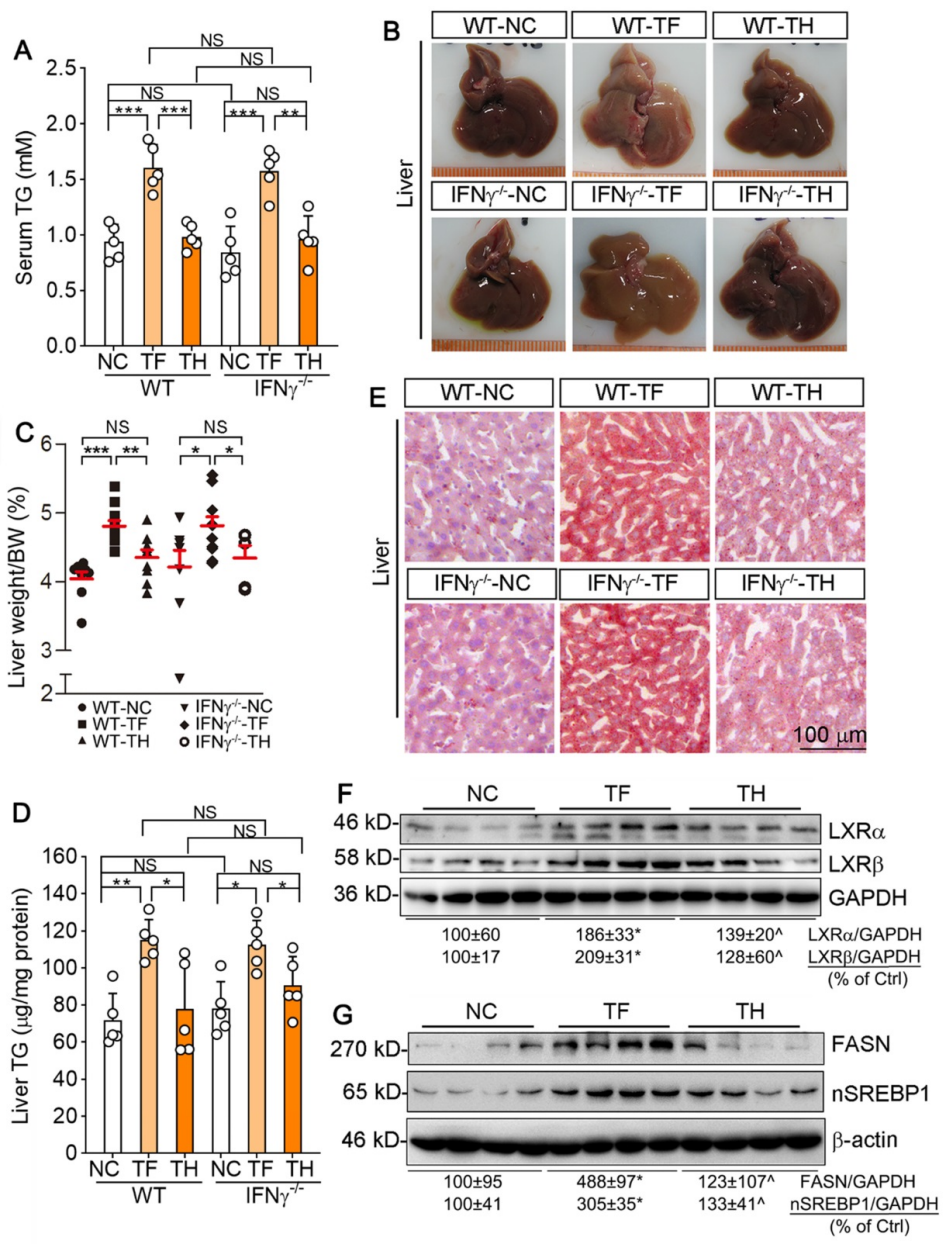
** D-Nap-GFFY-T317 blocks hypertriglyceridemia and fatty liver induced by the long-term of T317 treatment in both WT and IFNγ^-/-^ mice.** Blood and liver samples were collected from mice used in Figure 6, and conducted the following assays. (**A**) serum TG levels were determined using an assay kit. (**B-C**) liver was photographed, and weighed, followed by calculation of the ratio of liver weight to body weight. (**D-E**) lipid content in the liver was determined by Oil Red O staining of liver frozen sections and TG quantitative assay with total lipid extracted from a piece of liver (normalized by liver protein content). (**F-G**) expression of LXRα and LXRβ, FASN and nSREBP1 protein in the liver was determined by Western blot. (**A, C-D**): *P < 0.05, **P < 0.01, ***: P < 0.001; NS: not significantly different. n = 14 for WT mice, n ≥ 8 for IFNγ^-/-^ mice as indicated in Table 2; (**F-G**): *P < 0.05 vs. NC, ^P < 0.05 *vs*. TF, n = 4.

Indeed, we determined that T317 oral administration but not D-Nap-GFFY-T317 injection increased serum TG levels in both type of mice (Figure 8A). In the liver, T317 oral administration reduced liver color while increasing liver weight, but both were removed by D-Nap-GFFY-T317 injection (Figure 8B-C). Furthermore, D-Nap-GFFY-T317 injection attenuated T317-induced lipid accumulation and TG levels in both WT and IFNγ^-/-^ mice (Figure 8D-E). Mechanistically, the long-term T317 oral administration increased expression of LXRα and LXRβ in mouse liver, while D-Nap-GFFY-T317 injection had slight effect on both (Figure 8F). Consequently, expression of FASN and nSREBP1 was activated by T317 oral administration, not by D-Nap-GFFY-T317 injection (Figure 8G).

## Discussion

Lipid metabolism, especially the fatty acid synthesis, has been determined as a potential target for cancer treatment 24. Baron *et al.* has hypothesized that FASN, the key enzyme responsible for *de novo* fatty acid synthesis, as a potential oncogene 25. Although LXR activation can activate FASN expression/activity, many studies have demonstrated that LXR ligands can function as anti-tumorigenic reagents in multiple tumor models by the effects on cell cycle, tumor immunity and microenvironment 4, 26. Lin Z *et al.* has demonstrated that LXR activation potentiates sorafenib sensitivity in HCC by activating microRNA-378a transcription 27. In fact, the synthetic LXR agonists, such as T317 and GW3965, have been widely used in anti-atherosclerosis and tumor-related experimental studies 28-30. However, the side effects of the synthetic LXR agonists have not been paid enough attention. Therefore, finding effective LXR agonists that specifically act on macrophages is of great significance for anti-atherosclerosis and tumor therapy. Desmosterol and similar structure mimics, such as DMHCA and MePipHCA, stimulate LXR in macrophages while avoiding the activation of fatty acid synthesis triggered by SREBP 31. Yasuda *et al.* showed that use of the intestinal-specific LXR agonist GW6340 can prevent atherosclerosis by promoting the reverse transport of cholesterol in macrophages with little side effects on the liver 10. In addition, using nanoparticles to delivery LXR agonists specifically to macrophages is another way. Nanoparticles containing synthetic GW3965 can inhibit the development of atherosclerosis without causing liver steatosis 32. Guo *et al.* encapsulated T317 in synthetic HDL (sHDL) nanoparticles to increase cholesterol efflux 33. sHDL nanoparticles accumulate in the atherosclerotic plaques of apoe-deficient mice while avoiding liver lipid accumulation 33. In this study, we encapsulated T317 in D-Nap-GFFY hydrogel to promote the expression of IFNγ in macrophages for anti-tumor effect. This complex was not taken up by liver cells, thereby having no effect on hepatic lipogenesis, and demonstrating another novel approach to exert the positive effects of LXR agonist and remove its side effects efficiently.

IFNγ plays several important roles in tumor biology, such as inhibition of cell proliferation and angiogenesis, and induction of cell apoptosis in tumors 34. We previously identified IFNγ as a molecular target of LXR activation 12. Administration of LXR ligand increases IFNγ production in mice, particularly in macrophages. Therefore, treatment of LXR ligand to mice bearing either xenografted tumor cells or injected carcinogen increased animal survival or tumor-free rate 12. We further demonstrated the protection of LXR ligand against tumor growth depends on activation of IFNγ expression, indicating the critical role of cytotoxic effect of macrophage IFNγ on tumor cells 15. In this study, in addition to confirming anti-tumorigenic effect of T317, we employed a novel approach to deliver T317. Compared with free T317, the hydrogel encapsulated T317 demonstrated greater effect on induction of IFNγ expression in peritoneal macrophages (Figure 3D), indicating the hydrogel synergized the function of LXR on IFNγ expression. In addition, higher IFNγ levels in serum and BALF were observed in D-Nap-GFFY-T317-injected mice than the animals receiving T317 oral administration (Figure 2E, 7D-E). The more production of IFNγ by D-Nap-GFFY-T317 injection than T317 oral administration resulted in further inhibition of tumor growth (Figure 2B; 6B, F).

25-Hydroxycholesterol (25-HC), an oxysterol derived from cholesterol, is produced by action of cholesterol 25-hydroxylase (CH25H). 25-HC has been demonstrated antiviral functions 35-37, indicating its important role in immunology. A recent study also shows its important functions in anti-tumorigenesis 38. 25-HC inhibits the uptake of tumor-derived extracellular vesicles (TEV) and the metastasis of healthy cells after uptake of TEV. A correlation between low leukocyte CH25H levels and poor prognosis has been observed in melanoma patients. In animal model, lack of CH25H expression enhances TEV uptake, TEV-induced pre-metastatic niche and melanoma lung metastases. Interestingly, we have reported that CH25H is an LXR target gene also, and 25-HC activates CH25H expression in an LXR-dependent manner since 25-HC functions as an endogenous LXR ligand. Expression of CH25H and 25-HC production in macrophages can also be activated by interferons including IFNγ. In current study, the potent activation of macrophage IFNγ production by D-Nap-GFFY-T317 injection implies both 25-HC production and CH25H expression can also be enhanced, another potential anti-tumorigenic mechanism of LXR ligands.

It has to be admitted that oral medication is simple, economical and painless for patients. Subcutaneous administration may involve the risk of pain, infection and local irritation 39. It is necessary to pay attention to the injection site and the equipment used as clean and sterile as possible 39. Both oral administration and subcutaneous injection of T317 resulted in fatty liver (Figure S9), which suggests that the severe lipogenic effects of T317 are administration-independent. On the other hand, D-Nap-GFFY is a small molecule polypeptide that can be quickly digested after oral administration, making it difficult to achieve the purpose of slow release of drugs. Therefore, we encapsulated T317 using D-Nap-GFFY hydrogel and injected the complex of D-Nap-GFFY-T317 subcutaneously to exert the anti-tumorigenic effect of T317 and remove the side effect of T317. Indeed, after s.c. injection of D-Nap-GFFY-T317, we found that D-Nap-GFFY-T317 inhibited tumor growth and tumor angiogenesis better (Figure 2B, 7F), and had little effect on hepatic lipogenesis and fatty liver (Figure 5, 8), compared to T317 oral administration.

Compared with injection of hydrogel-encapsulated T317, the oral administration or injection of free T317 stimulates expression of FASN and SREBP1, thereby inducing *de novo* fatty acid biosynthesis, fatty liver and hypertriglyceridemia. The hydrogel-encapsulated T317 had no effect on expression of FASN and SREBP1 in the liver, thereby removing T317-induced fatty liver and hypertriglyceridemia.

Several studies have demonstrated the activation of immunity by hydrogels to improve anti-tumor therapy 40-43. The previous studies showed that D-Nap-GFFY functioned as a promising vaccine adjuvant since it can be selectively taken up by APCs including macrophages 17. In this study, we observed more induction of IFNγ production by D-Nap-GFFY-T317 injection than T317 oral administration, which may imply that APCs can take up more T317 from D-Nap-GFFY-T317 injection than T317 oral administration. Although D-Nap-GFFY-T317 had little effect on the liver or hepatocytes, in a separate study we observed that injection of D-Nap-GFFY-T317 is still able to act on Küpffer cells, the macrophages presenting in the liver, which may increase IFNγ expression locally. Furthermore, in this separate study, we determined the mechanism in which the selective uptake of D-Nap-GFFY-T317 by macrophages is mediated by scavenger receptor type A (SR-A) based on the following observations: 1) SR-A expression in macrophages is much higher than hepatocytes; 2) inhibition of SR-A expression, not other molecules involved in macrophage phagocytosis, such as CD14, CD47, CD11b and Ha-Aa, by siRNA reduced uptake of D-Nap-GFFY by macrophages; 3) inhibition of SR-A by siRNA also blocked induction of ABCA1 (ABCA1 is also a target of LXR) expression by D-Nap-GFFY-T317 in macrophages. Besides APCs, other immune cells, such as T cells, can also express IFNγ to suppress tumor growth. In this study, we determined that either T317 oral administration or D-Nap-GFFY-T317 injection enhanced infiltration of T cells in tumors associated with activated IFNγ expression (Figure 4A-B). However, T317 activated IFNγ expression is in LXR-dependent manner. Thus, expression profile of LXR can greatly influence the function of T317 on IFNγ expression. Compared with macrophages, it has been reported that expression of LXR, particularly LXRα, is at much lower levels 44. Therefore, we believe that APCs is the main source of IFNγ production in response to D-Nap-GFFY-T317 injection.

After s.c. injection, D-Nap-GFFY-T317 is selectively taken up by APCs, and protease in cells induces degradation of D-Nap-GFFY hydrogel, thereby releasing the encapsulated T317. Once released, T317 can activate LXR to induce IFNγ expression, a molecule can be secreted into circulation to act on remote cells or tissues. In addition, after uptake of D-Nap-GFFY-T317, APCs or monocytes can migrate/transport to remove target tissues/tumors before the complete release of T317. Therefore, we still observed that both LXR and IFNγ can be activated simultaneously by D-Nap-GFFY-T317 at both mRNA and protein levels in lymph nodes and lung (Figure 2H-I, 7F-G). Because the release of T317 in APCs is very slow and the released T317 can function these type cells in an autocrine-like manner, so a tiny T317 was determined in serum, compared with T317 oral administration (Figure 1H, J). Therefore, no direct effect of T317 on liver, such as lipid accumulation in the tissue, was observed.

In summary, injected D-Nap-GFFY-T317 nanofiber hydrogel can be exclusively taken up by APCs. Activation of IFNγ expression by T317 depends on expression of LXR, the ligand-activated transcription factor 45. More importantly, hepatocytes are not able to take up D-Nap-GFFY-T317. Therefore, injection of D-Nap-GFFY-T317 nanofiber hydrogel enhances the maturation and infiltration of DCs in tumors and activates IFNγ expression in both macrophages and DCs. The activated IFNγ has potent cytotoxic effect to tumor cells and inhibits angiogenesis in tumors, thereby reducing growth of xenografted or carcinogen-injected tumors without adverse effects on the liver and lipid metabolism. These findings indicate that the hydrogel-encapsulated LXR ligand might be a novel therapy for cancer treatment.

## Materials and methods

### Materials

T317 was purchased from Cayman Chemical (Ann Arbor, MI, USA). Rabbit anti-IFNγ, LXRα, LXRβ, CD11c, Ki-67 polyclonal antibodies and mouse anti-CD31 monoclonal antibody were purchased from Proteintech Group Inc. (Chicago, IL, USA). Rabbit anti-TTF-1, SPC, CD8a and VEGFR2 polyclonal antibodies were purchased from ABclonal Biotechnology Co., Ltd (Wuhan, Hubei, China). Rabbit anti-LY75 polyclonal antibodies were purchased from Abcam (Cambridge, MA). Mouse anti-CD68, CD3 monoclonal antibodies and rat anti-IFNγ monoclonal antibody were purchased from Santa Cruz Biotechnology, Inc. (Santa Cruz, CA, USA). FITC or APC-conjugated hamster anti-mouse CD11c and CD80 monoclonal antibodies were purchased from Sungene Biotech Biotechnology Co., Ltd (Shanghai, China). IFNγ ELISA assay kit was purchased from ABclonal Biotechnology Co., Ltd. The reverse transcription kit and the SYBR green PCR master mix were purchased from Vazyme Biotech Co., Ltd (Nanjing, China). D-Nap-GFFY was synthesized as described 17.

### General methods

The synthesized D-Nap-GFFY was characterized by ^1^H NMR and ^13^C NMR (Bruker ARX 400). Transmission electron microscope (TEM) samples (10 μL) were dried in a desiccator and then observed with Tecnai G2 F20 46. We performed rheological test on AR 2000ex with 40 mm parallel plates at a gap of 500 μm as described 47, 48. LC-MS was performed on Shimadzu LCMS-20AD (Japan) system.

### Cell culture

RAW264.7 cells and LLC1 cells were purchased from ATCC (Rockville, MD, USA) and cultured in RPMI 1640 medium supplemented with 10% fetal bovine serum (FBS), 50 mg/mL penicillin/streptomycin and 2 mM glutamine, respectively. RAW264.7 cells at ~90% confluence received treatment in serum-free medium. The thioglycollate-elicited peritoneal macrophages were collected from WT mice as described 49. All cells were incubated in a humidified atmosphere of 95% air and 5% CO_2_ at 37 °C.

DC 2.4 cells (a murine dendritic cell line) were purchased from Sigma-Aldrich (St. Louis, MO, USA) and cultured in DMEM medium supplemented with 10% FBS, 50 mg/mL penicillin/streptomycin and 2 mM glutamine, respectively.

Bone marrow cells (BMCs) were isolated by flushing from mouse femur with pre-cooled PBS, and cultured in RPMI 1640 medium supplemented with 10% FBS, 50 mg/mL penicillin/streptomycin and 2 mM glutamine, respectively. To induce differentiation of BMCs into DCs, after BMCs attached dishes well, half of the medium was replaced with fresh complete RPMI 1640 medium containing granulocyte-macrophage colony-stimulating factor (GM-CSF, 20 ng/mL) and interleukin 4 (IL-4, 10 ng/mL). After 6 days of culture, the immature DCs were collected, counted and re-distributed equally in 6-well culture plates for the subsequent experiments.

### Animals

The protocols for* in vivo* studies with mice were granted by the Animal Ethics Committee (No. HFUT-NKU20190215001C) and conforms to the Guide for the Care and Use of Laboratory Animals published by the National Institutes of Health (NIH Publications No.8023, revised 1978). IFNγ^-/-^ and WT mice on a C57BL/6 background were purchased from the Animal Center of Nanjing University (Nanjing, China). The animals were housed under specific pathogen-free conditions with free access to water and food at the Animal Center of Nankai University.

### Preparation of hydrogels

2 mg D-Nap-GFFY, 2 mg Na_2_CO_3_ and 1mL PBS (pH = 7.4) were added into a glass vial. The mixture was heated till the clear solution was obtained (final concentration of D-Nap-GFFY is 2 mg/mL), followed by cooling down to room temperature for hydrogel formation. To prepare D-Nap-GFFY-T317, we added indicated amount of T317 into the dissolved D-Nap-GFFY solution, vortexed to mix well and cooled down the solution to room temperature and form a hydrogel again. In order to clearly identify the changes of injected gel in mouse skin, the solvent of D-Nap-GFFY was changed from PBS to DMEM medium.

To determine the uniformity of D-Nap-GFFY, D-Nap-GFFY-T317 was centrifuged at 14,000 rpm for 15 min and the deposited gel was collected to determine T317 concentration at different locations of the gel by LC-MS.

### Release profiles of T317

The detection of drug release *in vitro* was performed as described 50. Briefly, 0.25 mL of PBS containing proteinase K at the indicated concentrations (0, 0.1 and 1 mg/mL) was added on the surface of D-Nap-GFFY-T317 and incubated at 37 °C. At each indicated time point, 0.2 mL of upper solution was collected and then 0.2 mL fresh PBS with the same concentrations of proteinase K was added. 100 μL of sample solution was used to measure the quantity of released T317 by LC-MS system and the release profiles of T317 were calculated as described 19.

The intracellular drug release behavior was determined with D-Nap-GFFY-encapsulated NR (100 ng/mL, D-Nap-GFFY-NR). DCs, primary peritoneal macrophages and HepG2 cells were incubated in the medium containing D-Nap-GFFY-NR for indicate times and the images were captured by a microscopy with an excitation wavelength at 480 nm.

### Determination of T317 in serum

C57BL/6 mice were randomly divided into 2 groups: one group received a single intragastric administration of T317 solution at a dose of 10 mg/kg bodyweight, and another group was s.c. injected D-Nap-GFFY-T317 with same dose of T317. After treatment, three mice in each group were anesthetized at the different time points (0, 1, 2, 4, 6, 8, 12 and 24 h) for blood collection and subsequently euthanized in a CO_2_ chamber. The blood samples were kept for 2 h at room temperature, centrifuged for 20 min at 2,000 g. The serum was transferred into a new test tube and T317 was extracted from serum as described for quantitative analysis by LC-MS 51.

### Determination of D-Nap-GFFY degradation

5 mL D-Nap-GFFY was treated with different concentrations of proteinase K at 37 °C. At the different time points, 150 μL of the solution was taken out, mixed with 150 μL of anhydrous methanol and analyzed remained D-Nap-GFFY with LC-MS.

C57BL/6 mice were s.c. injected 100 μL D-Nap-GFFY. At different time points, three mice were anesthetized and euthanized, then skin samples were collected followed by photography or HE staining.

### Tumor allograft experiment

LLC1 cells at ~80% confluence were collected. C57BL/6 (WT) or IFNγ^-/-^ mice were s.c. injected either 200 μL PBS or PBS containing 2 x 10^5^ LLC1 cells at the lateral axilla. After cell injection, WT or IFNγ^-/-^ mice were randomly divided into four groups and received the following treatment: a) NC group, mice were fed normal chow; b) H group, mice on normal chow were s.c. injected D-Nap-GFFY nanofiber hydrogel once another day; c) TF group, mice were fed normal chow containing T317 (5 mg/day/kg bodyweight); d) TH group, mice were fed normal chow and s.c. injected D-Nap-GFFY-T317 under the skin of their back once another day. Tumor volume was measured using calipers daily and calculated as described 12. At the end of the 18^th^ day after LLC1 cell injection, all the survival mice were anesthetized and euthanized in a CO_2_ chamber, followed by collection of blood, peritoneal macrophages and tissue (lung, liver and lymph nodes) samples individually.

To determine the mortality rate of mice inoculated with LLC1 cells, WT or IFNγ^-/-^ mice were randomly divided into three groups, mice in NC group were fed normal chow; mice in TF group were fed normal chow containing T317 (5 mg/day/kg bodyweight); mice in TH group were fed normal chow and s.c. injected D-Nap-GFFY-T317 (once every two days). Survival situation of these mice were checked and recorded daily.

### Urethane-induced lung adenocarcinoma model

WT or IFNγ^-/-^ mice were randomly divided into three groups. Mice in NC group were fed normal chow; mice in TF group were fed normal chow containing T317 (5 mg/day/kg bodyweight); mice in TH group were fed normal chow and s.c. injected D-Nap-GFFY-T317 once every two days. After one week of treatment, all mice were i.p. injected with urethane (1 g/kg bodyweight) once every 3 days for 8 times. At the end of 18^th^ week after the first urethane injection, all the mice were anesthetized and euthanized in a CO_2_ chamber, followed by collection of blood, peritoneal macrophages and tissue (BALF, lung, liver and lymph nodes) samples. The lung carcinoma incidence and the number of tumors on lung surface were recorded.

### Determination of serum lipid profiles and IFNγ levels

Blood samples were collected into a 1.5 mL tube and kept for 2 h at room temperature. Blood was centrifuged for 20 min at 2,000 g, and the serum was transferred into a new test tube and conducted the following assays. Levels of serum total cholesterol (T-CHO), HDL cholesterol (HDL-C), LDL cholesterol (LDL-C), TG, ALT, AST and ALP were measured using an automatic biochemical analyzer (Model 7020, Hitachi, Tokyo, Japan). Serum IFNγ levels were determined using mouse IFNγ ELISA kit according to the manufacture's instruction.

### Bronchioalveolar lavage

After euthanized, mouse lung was lavaged for 3 times with PBS, and the BALF was collected. BALF was then centrifuged for 10 min at 1,000 rpm. The supernatant was stored at -80 °C until determination of IFNγ levels using an ELISA kit according to the manufacture's protocols, while the pellet was re-suspended. Total cells in the suspension were counted using a grid hemocytometer, then conducted HE-staining for identification of different types of leukocytes. The numbers of different types of leukocytes were obtained by counting at least 500 cells on HE-stained cytocentrifuge slides.

### Determination of hepatic lipid content

For Oil Red O staining, the liver frozen sections were warmed at room temperature for 30 min and then soaked in PBS for 30 min. The sections were stained with Oil Red O solution (3 mg/mL in 60% isopropanol) for 45 min. After staining, sections were rinsed with 60% isopropanol, and then stained with alum haematoxylin solution for 30 sec for nuclei. Finally, sections were rinsed with distilled water and photographed by Leica DM3000 microscope (Wetzlar, Germany).

To quantitatively analyze TG content, ~30 mg of liver was homogenized in 1 mL 1 x PBS and then 100 μL homogenate was saved for determination of protein content which was used to normalize TG levels; 900 μL homogenate was used to extract total lipids followed by TG quantitative analysis as described 30.

### Hematoxylin and eosin (HE) and immunohistochemical (IHC) staining

Mouse skin and lung tissues were fixed in 4% paraformaldehyde, received water deprivation by standard procedures and embedded in paraffin and dissected into 5-μm-thick sections. Then the skin and lung sections were conducted HE or IHC staining as previously described 13. The average optical density (AOD) value of all the IHC images was calculated as described 52.

### Immunofluorescent (IF) staining

Mouse tumor samples were fixed in 4% paraformaldehyde, received water deprivation by 30% sucrose and embedded in OCT. The block was then dissected into 5-μm-thick sections, followed by IF staining as previously described 13. The mean fluorescence intensity (MFI) of all the IF images was calculated as described and presented either in main figures or supplemental figures due to space limitation 30.

### Western blot analysis

A piece of tissue (~30 mg) was homogenized in a lysis buffer 53. After centrifuge, the supernatant of homogenate was saved as tissue protein extract. Expression of LXRα, LXRβ, FASN, SREBP1 and IFNγ protein in tissue protein extract was determined by Western blot as described 54.

### Quantitative real time RT-PCR (qRT-PCR)

Total RNA was extracted from lymphatic tissue (~15 mg) as previously described 12. The cDNA synthesis was administrated with 500 ng total RNA by the reverse transcription kit. qRT-PCR was performed with SYBR green PCR master mix, and the primers listed in Table 4. Expression of LXRα, LXRβ, and IFNγ mRNA was normalized with GAPDH mRNA in the corresponding samples.

### Flow cytometry

To analyze expression of CD11c and CD80 protein on cellular surface, BM-DCs were collected and stained with FITC-conjugated CD11c, APC-conjugated CD80. Cells were then conducted fluorescence-activated cell sorting (FACS) assay as described 55.

### Statistical analysis

Values are expressed as indicated in the legend, and all experiments were repeated at least 3 times independently. At first, all the data were tested the normal distribution analysis with SPSS software. The data followed normal distributions were then analyzed by a one-way ANOVA/two-way ANOVA with Bonferroni post-test. Significant values were considered if P < 0.05 (n ≥ 3).

## Supplementary Material

Supplementary figures.

## Figures and Tables

**Table 1:** Impact of different treatment on serum parameters in LLC1-injected mice.

	Parameter					
Group	T-CHO	HDL-C	LDL-C	ALT	AST	ALP
WT-NC	2.41±0.56	1.04±0.31	0.21±0.05	15.1±2.3	141.3±12.9	69.3±9.0
WT-H	2.39±0.59	1.00±0.31	0.22±0.06	16.1±4.6	155.3±36.7	63.0±15.8
WT-TF	3.23±0.22**	1.38±0.16	0.32±0.08*	25.5±4.2***	205.1±65.3	96.4±15.8*
WT-TH	2.44±0.50^$^	1.29±0.15	0.22±0.06^$^	16.8±1.6^$^	156.3±6.0	68.7±16.7^$^
IFNγ^-/-^-NC	2.19±0.26	1.11±0.03	0.21±0.02	16.3±3.1	110.3±23.8	64.5±18.0
IFNγ^-/-^-H	2.17±0.37	1.16±0.15	0.22±0.02	16.7±3.5	144.0±12.3	61.3±12.0
IFNγ^-/-^-TF	3.36±0.24^#^	1.34±0.28	0.35±0.04^#^	25.0±1.4^#^	247.0±102.2	106.5±17.8^#^
IFNγ^-/-^-TH	2.36±0.45^¶^	1.22±0.13	0.23±0.04^¶^	17.3±1.3^¶^	166.4±42.6	66.4±16.8^¶^

Female wild-type and IFNγ^-/-^ mice received the treatment as indicated in Figure 2. Serum samples were collected and used to determine total cholesterol (T-CHO), HDL- and LDL-cholesterol (HDL-C, LDL-C) levels (mM); activity of ALT, AST and ALP (U/L). *P < 0.05, **P < 0.01 and ***P < 0.001 *vs*. WT-NC; ^#^P < 0.05 *vs.* IFNγ^-/-^-NC; ^$^P < 0.05 *vs.* WT-TF; ^¶^P < 0.05 *vs.* IFNγ^-/-^-TF; n = 10.

**Table 2:** The death rate of urethane-injected mice at the end of treatment.

Group	Total	Survived (w/tumors)	Survived (w/o tumors)	Died	Death rate
WT-NC	15	8	6	1	6.67%
WT-TF	15	5	9	1	6.67%
WT-TH	15	2	12	1	6.67%
IFNγ^-/-^-NC	11	5	3	3	27.27%
IFNγ^-/-^-TF	14	7	4	3	21.43%
IFNγ^-/-^-TH	12	6	3	3	25.00%

Female wild-type and IFNγ^-/-^ mice received the treatment as indicated in Figure 6A. Survived (w/tumors): the mice were still alive at the end of experiment but had tumors; Survived (w/o tumors): the mice were still alive at the end of experiment and had no tumors.

**Table 3:** Impact of different treatment on serum parameters in urethane-injected mice.

	Parameter					
Group	WT-NC	WT-TF	WT-TH	IFNγ^-/-^-NC	IFNγ^-/-^-TF	IFNγ^-/-^-TH
T-CHO	2.33±0.32	3.04±0.15***	2.43±0.32^$^	2.64±0.30	3.30±0.20^###^	2.52±0.34^¶^
HDL-C	1.47±0.28	1.53±0.16	1.54±0.17	1.50±0.22	1.54±0.28	1.49±0.42
LDL-C	0.21±0.06	0.38±0.08***	0.26±0.08^$^	0.24±0.10	0.36±0.12^###^	0.25±0.10^¶^
ALT	22.1±6.5	22.2±6.1	17.4±4.9^&^	20.0±8.9	22.6±5.7	18.8±5.2
AST	112.0±13.8	144.5±46.7*	108.5±16.0^$^	110.0±12.5	143.1±26.7^#^	121.7±17.9
ALP	115.5±52.1	136.5±25.8	78.6±24.5^&$^	109.3±51.2	114.8±36.3	91.8±30.9

Female wild-type and IFNγ^-/-^ mice received the treatment as indicated in Figure 6A. At the end of experiment, serum samples were collected from survived mice and used to detect T-CHO, HDL-C and LDL-C levels (mM), and activity of ALT, AST and ALP (U/L). *P < 0.05 and ***P < 0.001 *vs*. WT-NC; ^#^P < 0.05 and ^###^P < 0.001 *vs.* IFNγ^-/-^-NC; ^&^P < 0.05 *vs.* WT-NC; ^$^P < 0.05 *vs.* WT-TF; ^¶^P < 0.05 *vs.* IFNγ^-/-^-TF; n ≥ 8.

**Table 4:** Sequences of primers for qRT-PCR analysis.

Gene*	Sense	Anti-sense
LXRα	CTCAATGCCTGATGTTTCTCCT	TCCAACCCTATCCCTAAAGCAA
LXRβ	ATGTCTTCCCCCACAAGTTCT	GACCACGATGTAGGCAGAGC
IFNγ	GAGGAACTGGCAAAAGGATGGTGA	TTTGTTGCTGATGGCCTGATTGT
GAPDH	TGTGTCCGTCGTGGATCTGA	TTGCTGTTGAAGTCGCAGGAG

*: LXRα, liver X receptor α; LXRβ, liver X receptor β; IFNγ, interferon-γ; GAPDH, glyceraldehyde 3-phosphate dehydrogenase.

## Abbreviations

25-HC: 25-hydroxycholesterol
AAH: atypical adenomatous hyperplasia
AD: adenomas
ALP: alkaline phosphatase
ALT: alanine aminotransferase
APCs: antigen-presenting cells
AST: aspartate aminotransferase
BALF: bronchoalveolar lavage fluid
BMCs: bone marrow cells
CH25H: cholesterol 25-hydroxylase
CTLs: cytotoxic T lymphocytes
DCs: dendritic cells
D-Nap-GFFY: naphthylacetic acid modified D-enantiomeric-glycine-phenylalanine-phenylalanine-tyrosine
FACS: fluorescence-activated cell sorting
FASN: fatty acid synthase
FBS: fetal bovine serum
GM-CSF: granulocyte-macrophage colony-stimulating factor
IFNγ: interferon gamma
IL-4: interleukin 4
LLC1: Lewis lung carcinoma
LXR: liver X receptor
NR: Nile red
OVA: ovalbumin
SPC: surfactant protein C
SREBP1: sterol-responsive element binding protein 1
T317: T0901317
TEV: tumor-derived extracellular vesicles
TEM: transmission electron microscope
TG: triglyceride
TTF-1: thyroid transcription factor 1
