# Supplementary material for: Encapsulation of LXR ligand by D-Nap-GFFY hydrogel enhances anti-tumorigenic actions of LXR and removes LXR-induced lipogenesis
Source: Theranostics. 2021 Jan 1;11(6):2634–54. doi: 10.7150/thno.53139 (PMC7806465; doi:10.7150/thno.53139)
Supplement: Supplementary file 1 — Supplementary figures. [file thnov11p2634s1.pdf]

Supporting Information

Encapsulation of LXR ligand by D-Nap-GFFY hydrogel enhances anti-tumorigenic actions of LXR and removes LXR-induced lipogenesis

Ke Feng, Chuanrui Ma, Yuxin Liu, Xiaoxiao Yang, Zhimou Yang, Yaoxia Chen, Tengyan Xu, Chengbiao Yang, Shuang Zhang, Qi Li, Zhuo Wei, Dan Zhao, Peng Zeng, Jihong Han, Jie Gao, Yuanli Chen , Yajun Duan

Synthesis and characterization of D-Nap-GFFY and D-Nap-GFFY-T317

Synthesis of D-Nap-GFFY

The peptides were prepared by the standard Fmoc solid phase peptide synthesis (SPPS) method using 2-chlorotrityl chloride resin and the corresponding N-Fmoc protected amino acids protected by side chains. The first amino acid was loaded on resin at the C-terminal with a loading efficiency of about 1.3 mmol/g. The 20% piperidine was used to protect the Fmoc group in anhydrous N,N'-dimethylformamide (DMF). Then, O-(Benzotriazol-1-yl)-N,N,N',N'-tetramethyluroniumhexafluorophosphate (HBTU) was used as a coupling agent, the next Fmoc-protected amino acid was coupled to a free amino group. The growth of the peptide chain was carried out according to the established Fmoc SPPS protocol. In the last step, the amine group of the peptide was capped with 2-naphthaleneacetic acid (Nap) or another aromatic blocking group. After the last coupling step, the excess reagent was removed by washing with dimethylformamide (DMF) for 5 times (5 mL per gram of resin), followed by washing with dichloromethane (DCM) for 5 times (5 mL per gram of resin). In order to cleave the peptide derivatives from the resin, ice-cold 95% trifluoroacetic acid (TFA) was added, used, the

mixture of peptide/resin was stirred, filtered at room temperature, and finally poured into ice-cold diethyl ether. The resulting pellet was centrifugated for 10 min at 6,000 rpm and 4 °C. Afterwards the supernatant was removed, the solid was dried by a vacuum pump.

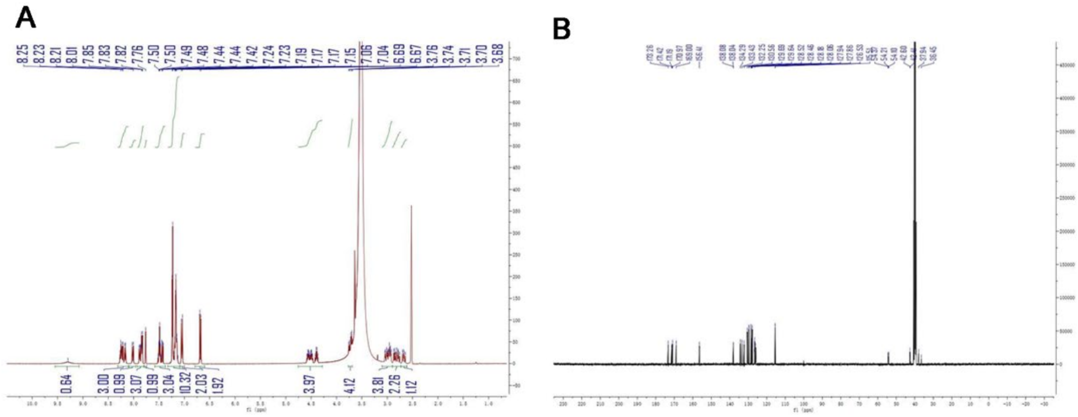

Figure S1. The ^{1}H and ^{13}C NMR of D-Nap-GFFY.
(**A**) ^{1}H NMR (400 MHz, DMSO) δ 9.29 (1 H, s), 8.35 – 8.09 (5 H, m), 8.02 (2 H, d, J 8.4), 7.84 (7 H, ddd, J 32.7, 18.4, 15.5), 7.63 – 7.32 (5 H, m), 7.31 – 7.10 (17 H, m), 7.05 (3 H, d, J 8.5), 6.68 (3 H, d, J 8.4), 4.66 – 4.45 (4 H, m), 4.39 (2 H, dd, J 13.5, 7.9), 3.78 – 3.68 (8 H, m), 3.09 – 2.90 (6 H, m), 2.89 – 2.72 (4 H, m), 2.67 (2 H, dd, J 13.9, 9.6). (**B**) ^{13}C NMR (101 MHz, DMSO) δ 173.26, 171.93 – 170.30, 169.00, 156.41, 138.06, 134.29, 133.43, 132.25, 130.56, 129.66, 128.49, 128.25 – 127.65, 126.72, 126.53, 126.01, 115.51, 55.26 – 52.10, 42.51, 37.94, 36.45.

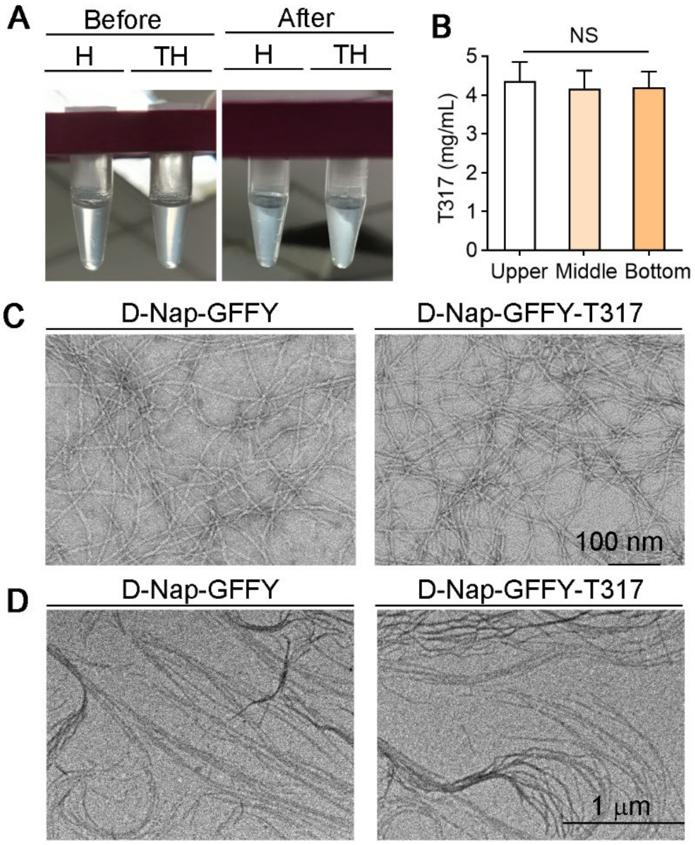

Figure S2. Photographs of D-Nap-GFFY and D-Nap-GFFY-T317.
(**A**) photographs of D-Nap-GFFY hydrogel prepared with PBS (H) and D-Nap-GFFY-T317 hydrogel prepared with PBS (TH) before and after centrifugation. (**B**) the concentration of T317 in each position of the hydrogel after centrifugation. (**C-D**) the images of D-Nap-GFFY and D-Nap-GFFY-T317 hydrogel by TEM study. Figure S2**C** is the original picture of Figure 1**C**.

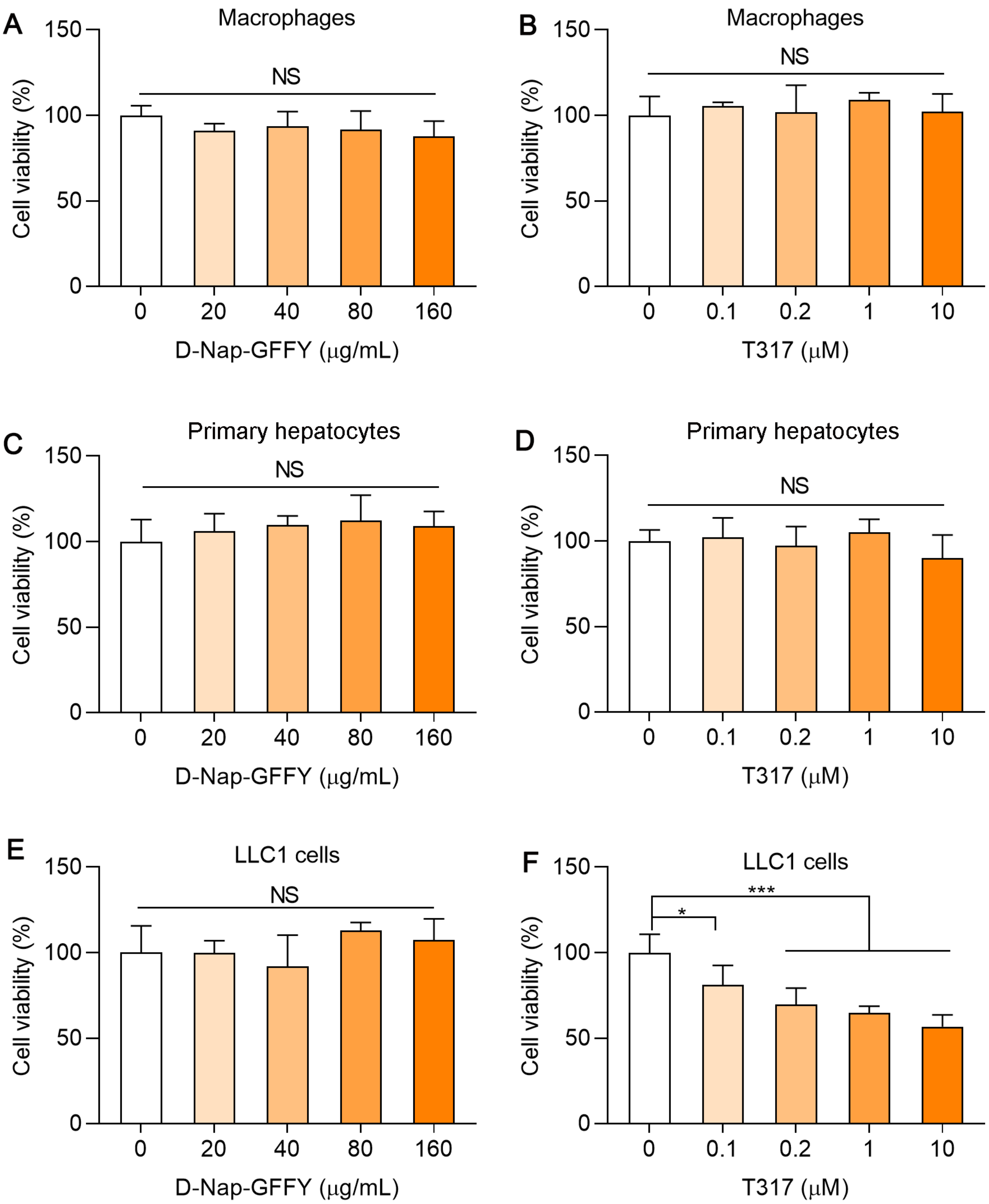

Figure S3. The effect of D-Nap-GFFY or T317 on cell viability of macrophages, hepatocytes and LLC1 cells. Peritoneal macrophages (**A-B**), primary hepatocytes (**C-D**) and LLC1 cells (**E-F**) in 96-well plates were treated with D-Nap-GFFY or T317 at the indicated concentrations for 16 h. Cell viability was determined using the CCK-8 method (n = 5).

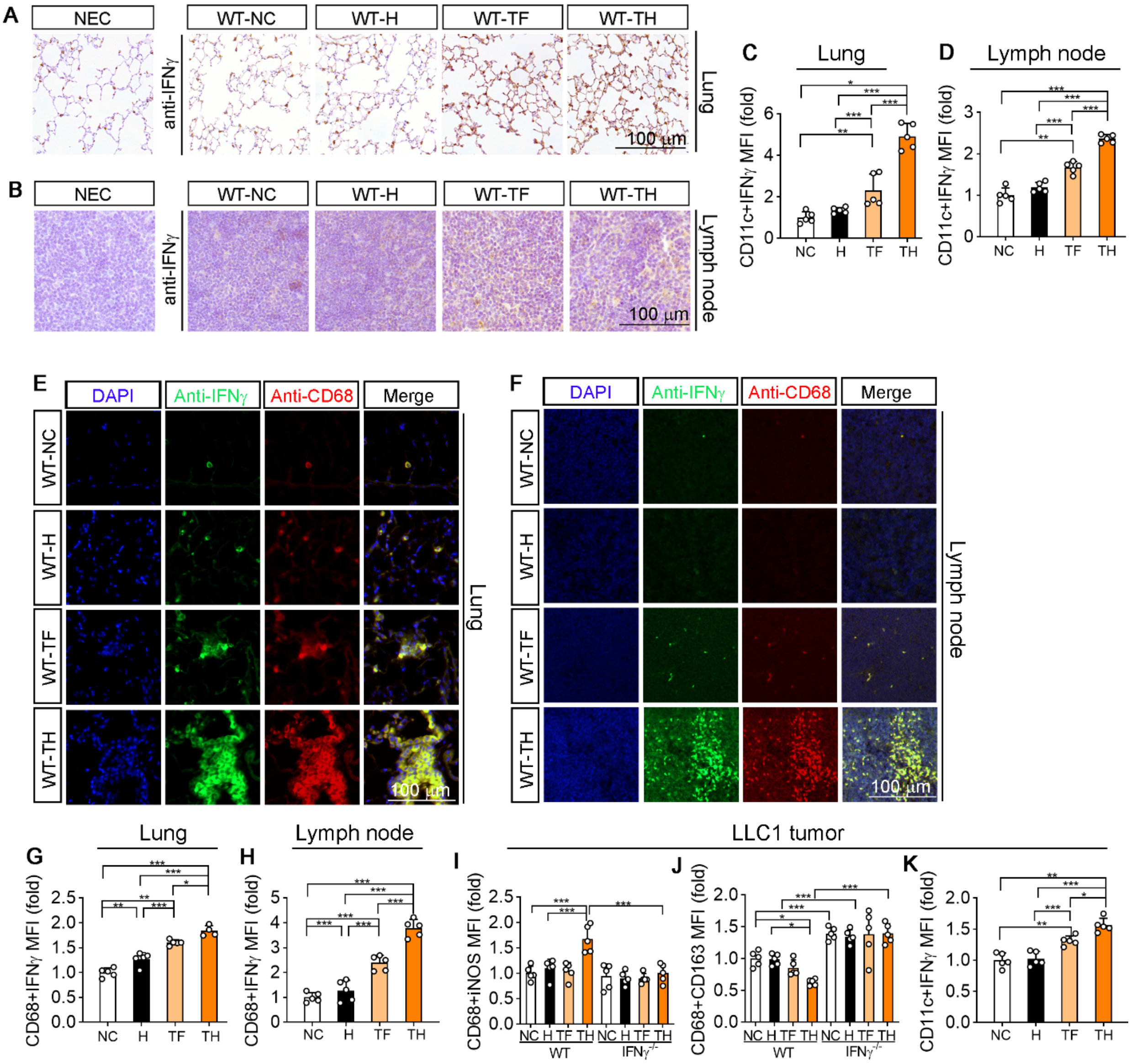

Figure S4. D-Nap-GFFY-T317 activates IFNγ expression and promotes APC infiltration in LLC1-inoculated mice. (**A-B**) expression of IFNγ protein in lung and lymph nodes was determined by IHC staining. NEC: negative control, normal IgG was used to replace primary antibody. (**C-D**) the MFI of images in Figure 2**F, G** was quantified. (**E-H**) co-immunofluorescent staining of lung and lymph node sections with anti-IFNγ and CD68 antibodies with quantitative analysis of MFI in images. (**I-J**) the MFI of images in Figure 3**E** was quantitaively analyzed. (**K**) the MFI of images in Figure 3**F** was quantitaively analyzed. The values of MFI were expressed as folds of the control group. *P < 0.05, **P < 0.01; ***P< 0.001, n = 5.

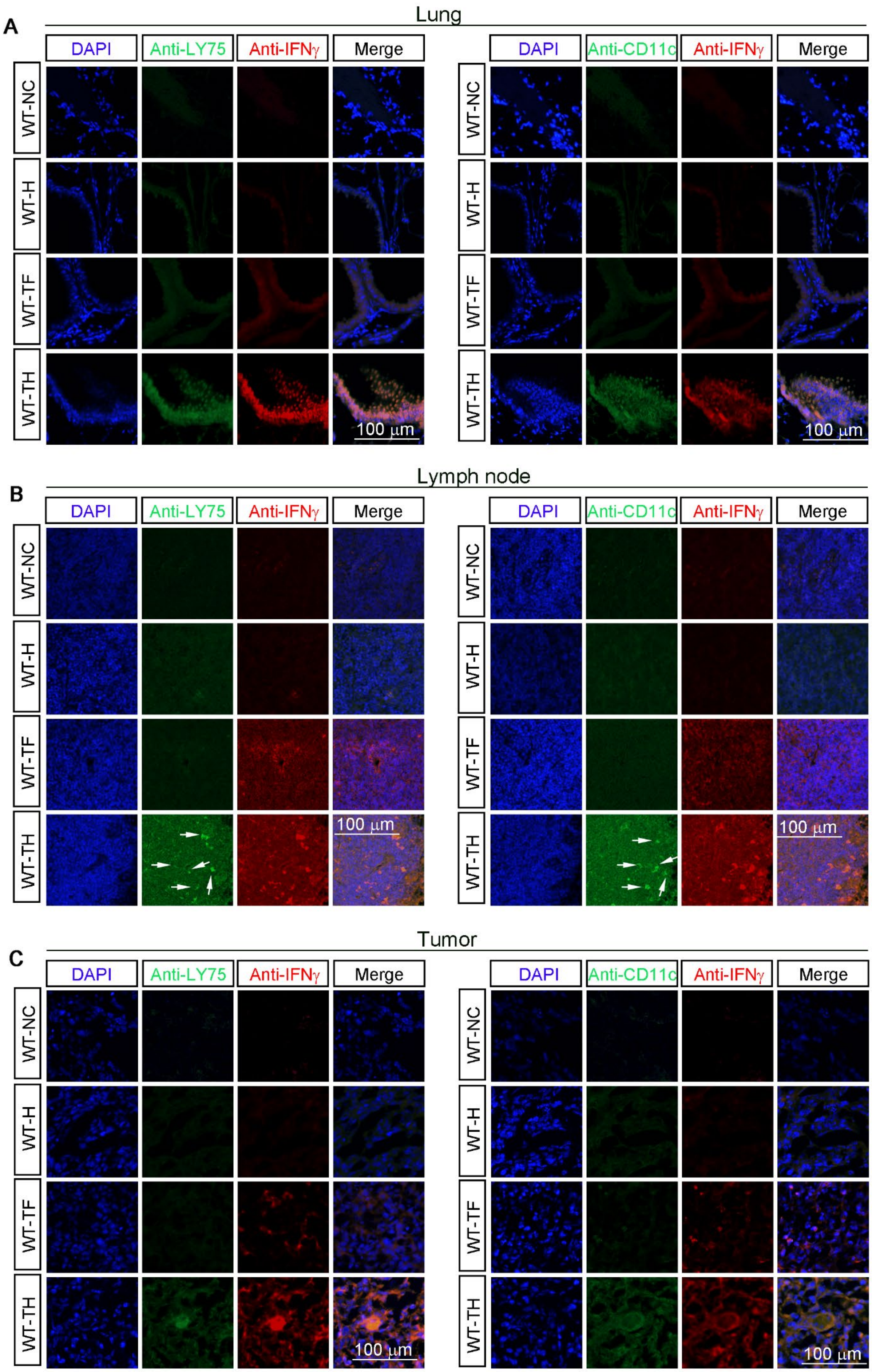
A
Lung
DAPI
Anti-LY75
Anti-IFNγ
Merge
Anti-CD11c
WT-NC
WT-H
WT-TF
WT-TH
100 μm
B
Lymph node
C
Tumor

Figure S5. Determination of DCs in tumor, lung and lymph nodes by co-immunofluorescent staining with anti-LY75 or CD11c and IFNγ antibodies in mice inoculated with LLC1 cells. The adjacent sections of lung (**A**), lymph node (**B**) and tumor (**C**) of mice used in Figure 2**B-D** were conducted co-immunofluoresent staining with anti-LY75 and IFNγ antibodies, or anti-CD11c and IFNγ antibodies to determine DCs. The arrows indicate DCs ($LY75^{+}CD11c^{+}$) in lymph node sections.

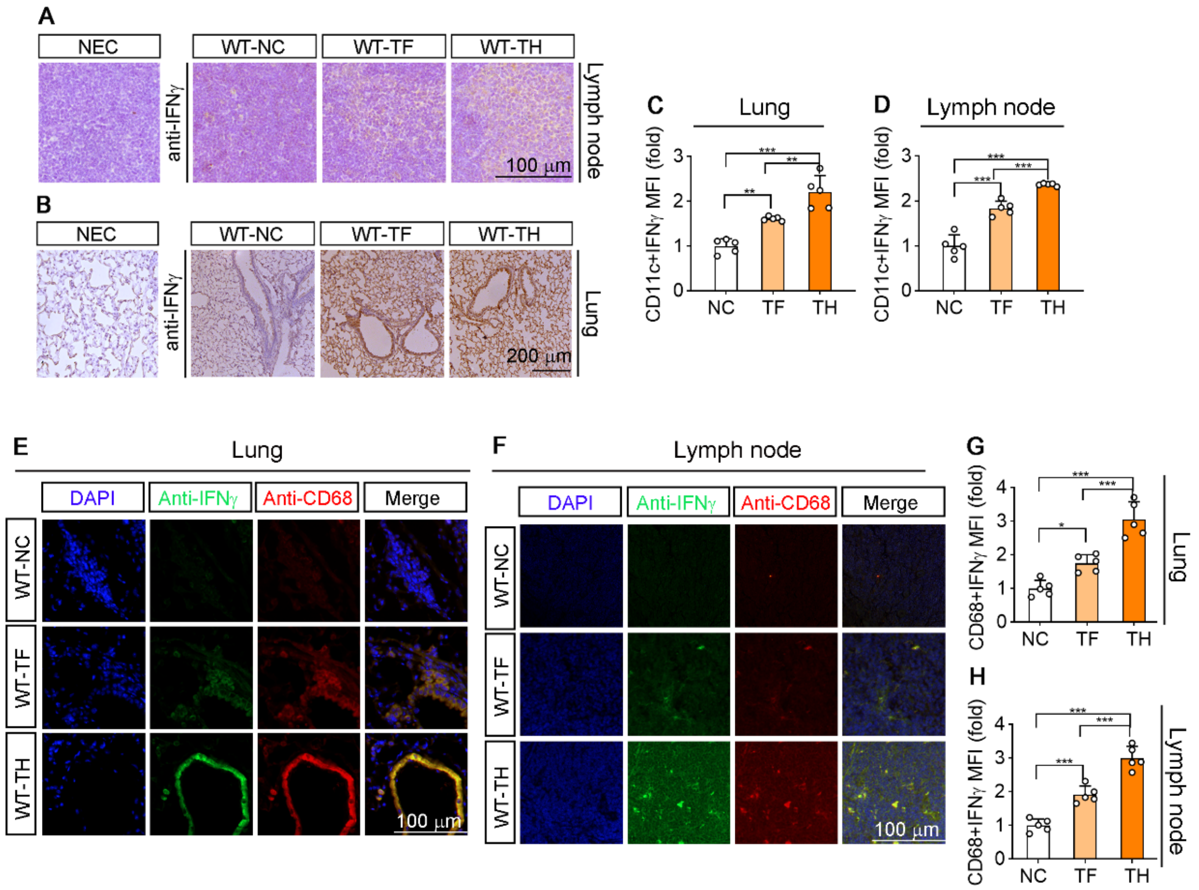

Figure S6. D-Nap-GFFY-T317 activates IFNγ expression and promotes infiltration of APCs into lymph node and lung in urethane-injected mice. At the end of experiment, both lung and lymph node samples were collecetd from mice used in Figure 6, followed by preparatio of tissue sections for the following assay. (**A-B**) expression of IFNγ protein in mouse lymph node and lung was determined by IHC staining. NEC: negative control, normal IgG was used to replace primary antibody. (**C-D**) the MFI of images of Figure 7**I-J** was quantified. (**E-H**) co-immunofluorescent staining of lung and lymph node sections with anti-IFNγ and CD68 antibodies with quantitative analysis of MFI of images. The values of MFI were expressed as folds of the NC group.*P < 0.05, **P < 0.01; ***P < 0.001, n = 5.

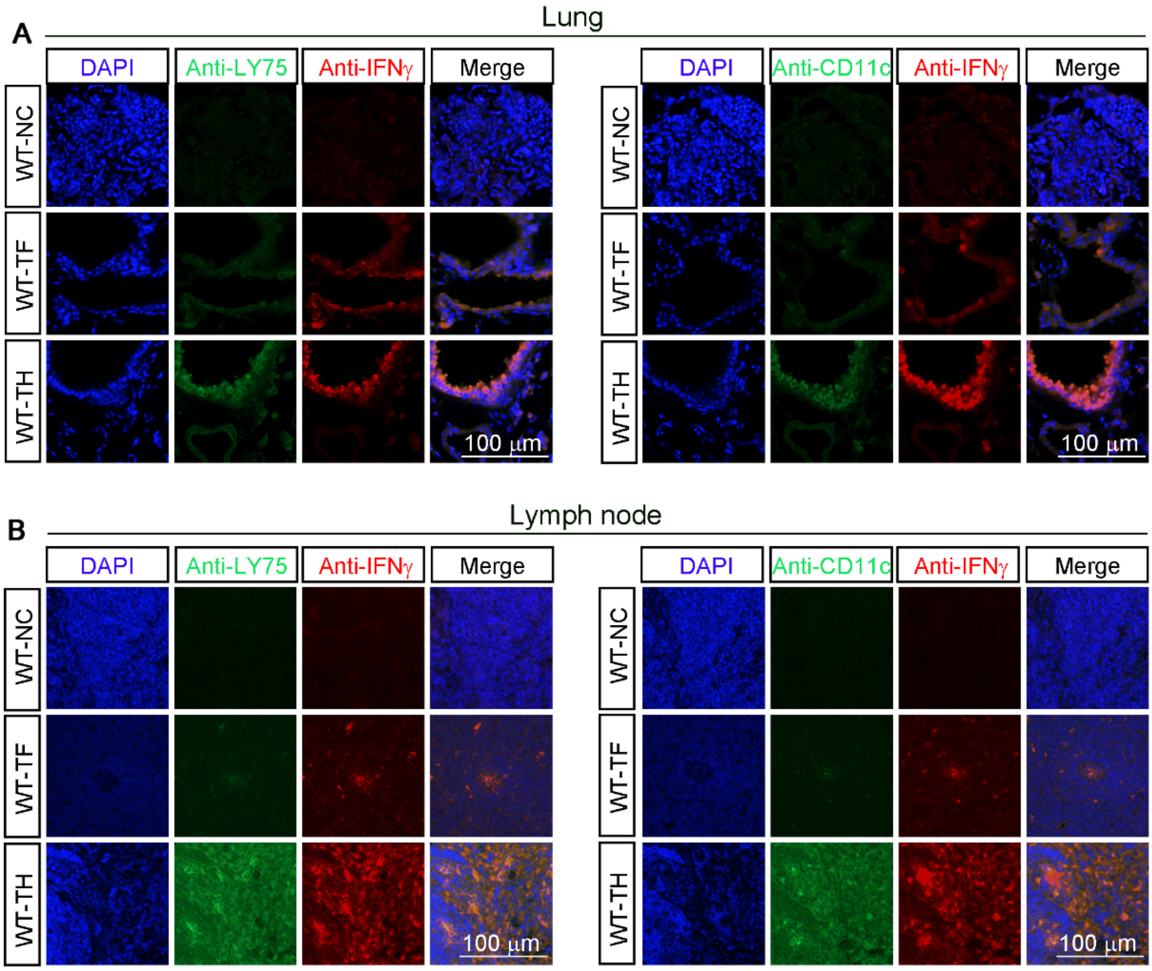

Figure S7. Co-immunofluorescent staining of lung and lymph node sections with anti-LY75, IFNγ and CD11c antibodies for determination of DCs in the urethane-injected mice. The adjacent sections of lung (**A**) and lymph nodes (**B**) of the mice used in Figure 6 were conducted co-immunofluoresent staining with anti-LY75 and IFNγ antibodies, or anti-CD11c and IFNγ antibodies to determine DCs.

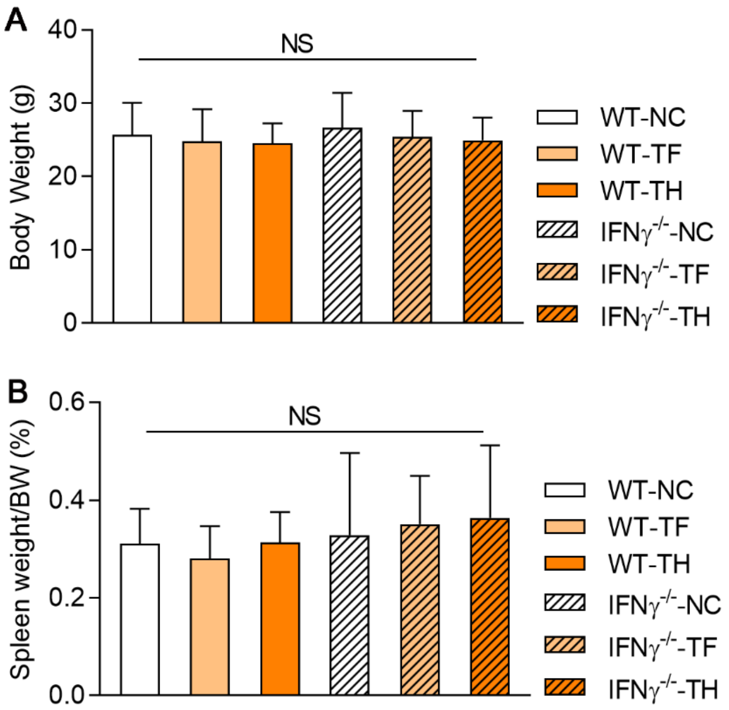

Figure S8. T317 oral adminstration or D-Nap-GFFY-T317 injection has no effect on urethane-injected mouse body weigh or the ratio of spleen weight to body weight. Female wild-type and IFNγ$^{-/-}$ mice received the treatment as indicated in Figure 6**A**. Body weight (**A**) and the ratio of spleen weight to body weight (**B**) were detected.

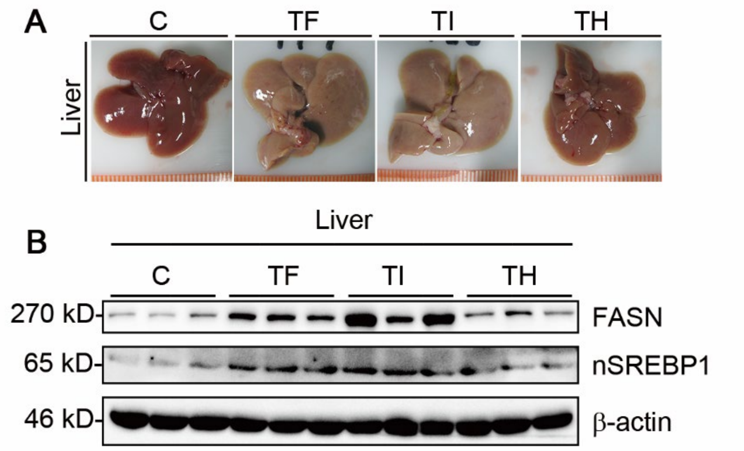

Figure S9. D-Nap-GFFY-T317 protects B16-treated WT mice against oral administration and subcutaneous injection of T317-induced hepatic lipid accumulation. After s.c. injection of B16 cells (2 x 10^5 cells/mouse), wild-type (WT) mice were randomly divided into 4 groups (10 mice/group) and received following treatment: C group, feeding normal chow plus s.c. injection of PBS; TF group, feeding normal chow containing T317 (5 mg/day/kg bodyweight); TI group, feeding normal chow plus s.c. injections of T317 dissolved in olive oil once another day with dose of T317 at 10 mg/kg bodyweight or equivalent to ~5 mg/day/kg bodyweight; TH group, feeding normal chow plus s.c. injections of D-Nap-GFFY-T317 once another day with dose of T317 at 10 mg/kg bodyweight or equivalent to ~5 mg/day/kg bodyweight. After 30 days, mice were euthanized and liver samples were collected for following assay. (**A**) liver photos. (**B**) expression of FASN and nSREBP1 protein in total protein extrated from a piece of liver was determined by Western blot.
